# A screening method to identify efficient sgRNAs in Arabidopsis, used in conjunction with cell-specific lignin reduction

**DOI:** 10.1186/s13068-019-1467-y

**Published:** 2019-05-23

**Authors:** Yan Liang, Aymerick Eudes, Sasha Yogiswara, Beibei Jing, Veronica T. Benites, Reo Yamanaka, Clarabelle Cheng-Yue, Edward E. Baidoo, Jenny C. Mortimer, Henrik V. Scheller, Dominique Loqué

**Affiliations:** 1 Joint BioEnergy Institute, Emeryville, CA 94608 USA; 2 Environmental Genomics and Systems Biology Division, Lawrence Berkeley National Laboratory, Berkeley, CA 94720 USA; 3 Biological Systems Engineering Division, Lawrence Berkeley National Laboratory, Berkeley, CA 94720 USA; 4 School of Public Health, University of California, Berkeley, CA 94720 USA; 5 Department of Plant and Microbial Biology, University of California, Berkeley, CA 94720 USA

**Keywords:** CRISPR/Cas9, Guide RNA efficiency, sgRNA, Constrained editing, HCT, GONST2, Genome editing, Lignin, Arabidopsis

## Abstract

**Background:**

Single guide RNA (sgRNA) selection is important for the efficiency of CRISPR/Cas9-mediated genome editing. However, in plants, the rules governing selection are not well established.

**Results:**

We developed a facile transient assay to screen sgRNA efficiency. We then used it to test top-performing bioinformatically predicted sgRNAs for two different Arabidopsis genes. In our assay, these sgRNAs had vastly different editing efficiencies, and these efficiencies were replicated in stably transformed Arabidopsis lines. One of the genes, *hydroxycinnamoyl*-CoA *shikimate/quinate hydroxycinnamoyltransferase* (*HCT*), is an essential gene, required for lignin biosynthesis. Previously, HCT function has been studied using gene silencing. Here, to avoid the negative growth effects that are due to the loss of HCT activity in xylem vessels, we used a fiber-specific promoter to drive CAS9 expression. Two independent transgenic lines showed the expected lignin decrease. Successful editing was confirmed via the observation of mutations at the *HCT* target loci, as well as an approximately 90% decrease in HCT activity. Histochemical analysis and a normal growth phenotype support the fiber-specific knockout of *HCT*. For the targeting of the second gene, *Golgi*-*localized nucleotide sugar transporter2* (*GONST2*), a highly efficient sgRNA drastically increased the rate of germline editing in T1 generation.

**Conclusions:**

This screening method is widely applicable, and the selection and use of efficient sgRNAs will accelerate the process of expanding germplasm for both molecular breeding and research. In addition, this, to the best of our knowledge, is the first application of constrained genome editing to obtain chimeric plants of essential genes, thereby providing a dominant method to avoid lethal growth phenotypes.

**Electronic supplementary material:**

The online version of this article (10.1186/s13068-019-1467-y) contains supplementary material, which is available to authorized users.

## Introduction

Several biotechnological approaches to improve biomass composition for downstream processing have been used in the past. Lignin is the most important contributor to biomass recalcitrance, but lignin-deficient mutants are generally deficient in growth. We have previously addressed that using vessel-specific complementation of a lignin biosynthesis mutant, whereby lignin was reduced in the fiber cell walls but maintained in the vessels, where lignin is critical [1]. A similar approach has been used to obtain plants with reduced xylan content or modified lignin composition [2–5]. This approach requires transformation of an identified mutant and can be difficult to apply to a range of bioenergy crops. We therefore wanted to develop methods based on dominant gene constructs that can be introduced into plants that do not already contain mutations in the pathways of interest. In this study, we have explored the use of clustered regularly interspaced short palindromic repeats/CRISPR-associated protein 9 (CRISPR/Cas9) genome editing to target genes of interest in a tissue-specific manner.

Endonuclease Cas9 or its orthologues [6, 7] form a complex with the guide RNA and cleave the target DNA. The resulting DNA double-strand break is repaired by one of two cellular repair mechanisms. Nonhomologous end joining (NHEJ) causes primarily random mutations at the site of the DNA double-strand break. Under certain conditions, homology-directed repair (HDR) may occur, allowing more genomic alteration events including DNA or gene deletion, insertion, replacement, etc. Efficacy of CRISPR/Cas9-mediated genome editing in plants has been demonstrated in a large number of studies [8].

For tissue-specific genome editing, it is essential to use highly efficient guide RNAs. Modified from the original crRNA–tracerRNA dual RNA complex in bacteria, the most frequently used guide RNA is the single guide RNA (sgRNA), which is composed of a target recognition sequence of 20 nucleotides (nt), also called a spacer sequence, and a backbone scaffold sequence, which forms the proper secondary structure for Cas9 binding [9, 10]. The length of spacer sequence and variation in sgRNA backbone sequence can impact sgRNA specificity and efficiency [11–13]. To reduce complication, in this study, we only refer to sgRNAs sharing the common backbone scaffold but differing in spacer sequences, which are restricted to 20–21 nt. For CRISPR editing, a short DNA sequence, named the protospacer adjacent motif (PAM) and often located 3–4 nt downstream of the cleavage site, is required for CAS protein recognition and cleavage. In the CRISPR editing system with Cas9 from *Streptococcus pyogenes*, the canonical PAM is 5′-NGG-3′ where “N” is any nucleobase followed by two guanine nucleobases (“G”), becoming the only defined restriction for sgRNA selection [9, 14]. Although the choice of sgRNA would seem to be unlimited, studies in various organisms indicated that the choice of sgRNA significantly affects the efficiency of CRISPR/Cas9 targeting and/or DNA cleavage at target loci [15–22]. In plants, the impact of sgRNA choice on CRISPR/Cas9 editing efficiency has been documented [23–25], but it is still unclear how to select an optimal sgRNA.

In the current study, we developed an assay to evaluate the editing efficiency of sgRNAs with various spacer sequences targeting the same gene of interest (GOI) based on a transient expression system in tobacco. These sgRNA sequences had all been identified as “high-quality” by multiple existing bioinformatic tools. The sgRNAs were coexpressed with the CRISPR/Cas9 protein to evaluate their potential editing efficiency. The best performers, alongside some of the poorer performers, were then tested in a stable transgenic system. One of our target genes, hydroxycinnamoyl-CoA shikimate/quinate hydroxycinnamoyltransferase (*HCT*, At5g48930), encodes a key enzyme in lignin biosynthesis [26]. We targeted the editing in a cell-specific manner to fiber cells, avoiding vessels. The resulting chimeric plants had normal growth properties while lignin was significantly reduced. The method used to select efficient guide RNAs is broadly applicable for plant engineering and not restricted to use for biomass improvement.

## Results

### Development of a facile system for assaying sgRNA efficiency

**Fig. 1:**
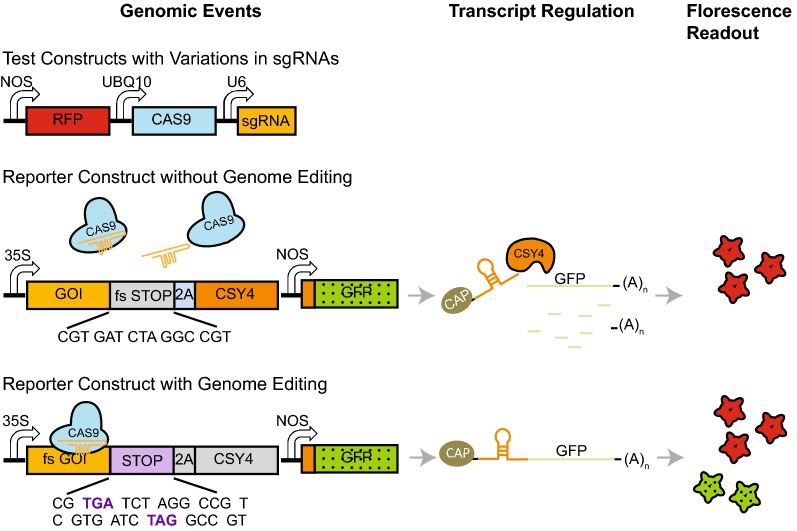
Development of a transient assay to test sgRNA efficiency for CRISPR editing in vivo. Model showing assay components and their predicted interactions. The symbols for the corresponding genomic sequence, transcript sequence, and protein product of the same gene are drawn in the same color. Gray designates deactivated modules in the reporter construct before or after CRISPR editing events. GOI: gene of interest; fs: frame-shifted; 2A: 2A peptide

Plants have long generation times, so inefficient sgRNAs selection can set back research by months or years, as well as wasting resources. We wanted to develop a system for prescreening sgRNAs using a tobacco (*Nicotiana benthamiana*) transient expression system. Our system is based on a transcript repression system previously developed in our laboratory [27]. This consists of a reporter construct and a test construct, both of which contain stacked genes to be expressed in tobacco leaves through *Agrobacterium tumefaciens* (Agrobacterium)-mediated transformation via leaf-infiltration (Fig. 1).

The reporter construct contains a gene of interest (GOI), either the open reading frame or the full genomic DNA, driven by a constitutive 35S promoter, and the reporter cogGFP driven by a constitutive NOS promoter. The “cog” in cogGFP designates a specific recognition site of Csy4 endoribonuclease for posttranscriptional cleavage [27]. The stop codon of the GOI is removed, and a linker sequence containing a frame-shifted Stop Codon (fs STOP) cassette is inserted between the GOI and the 2A peptide encoding sequence [28] fused to the coding sequence of *Csy4* [29]. This design means that a frame-shift in the GOI resulting from an editing event would prevent translation of Csy4.

The test construct contains a plant codon-optimized *Cas9* gene [30] driven by the constitutive *Ubquitin10* (*UBQ10*) promoter, and the sgRNA driven by the class III *U6* promoter from Arabidopsis. The sgRNA is composed of the spacer sequence to be tested, followed by a common sgRNA scaffold sequence. The test construct also encodes a pNOS::RFP cassette, which allows normalization of tobacco leaf transformation efficiency.

When the reporter construct is transformed alone, or together with a test construct containing a nontargeting sgRNA, the in-frame p35S::GOI::2A::Csy4 DNA construct enables the expression of the Csy4 protein. The Csy4 protein subsequently de-caps the cogGFP transcript through cleavage, resulting in repression of cogGFP expression [27] (Fig. 1). In contrast, if a test construct contains an active sgRNA, which triggers DNA cleavage by Cas9, single nucleotide insertion or deletions (indels) are most likely to form in place of the double strand break by NHEJ repair. Most indels cause a frame shift within the GOI and generate stop codons within the GOI. Alternatively, there is a 2/3 chance that the frame shift converts the fsSTOP cassette to an in-frame stop codon (STOP). In either case, the translation of Csy4 protein is eliminated, alleviating cogGFP repression. Therefore, GFP expression (once normalized to the RFP signal) will be positively correlated with the editing efficiency of the selected sgRNA.

### Testing the assay

**Fig. 2:**
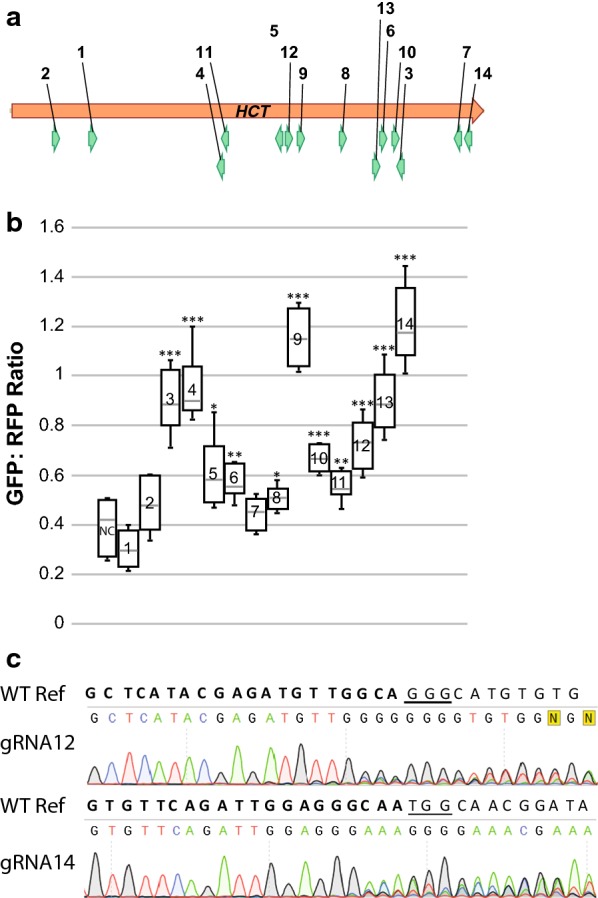
Editing efficiency analysis on 14 HCT_gRNAs. **a** Location of sgRNA target sites in the *HCT* gene. The open reading frame of the *HCT* gene is represented with an orange arrow. The binding site and direction of individual HCT_gRNA are represented with green arrows. **b** The relative efficiency of individual HCT_gRNA is expressed as GFP:RFP ratios. NC: negative control. Data represent 5–7 biological replicates. For details of the individual sgRNAs, see Additional file 1. Asterisks indicate significant differences compared to NC (Student’s *t*-test; *P < 0.05; **P < 0.01; ***P < 0.001). **c** Confirmation of mutations at the HCT_gRNA12 and HCT_gRNA14 target sites. Sequences spanning the target sites on *HCT* transcript were amplified and analyzed by Sanger sequencing. The unmodified (WT) sequence of the corresponding region is shown on top of the chromatogram with the spacer sequences in bold and the PAM sequences underlined

We applied a widely used bioinformatics tool, CRISPR-PLANT (http://www.genome.arizona.edu/crispr/CRISPRsearch.html), to identify candidate spacer sequences for targeting the *HCT* gene [31, 32]. A total of 146 sgRNA spacer sequences were identified to be Class 0.0 or Class 1.0 spacer sequences, i.e., they were predicted to specifically target *HCT* with low off-target potentials. From the 146 candidate spacers, 14 spacer sequences which targeted different regions along the *HCT* gene were selected for testing in our assay (Fig. 2a; Additional file 1) and used to generate 14 test constructs (HCT_gRNA1 to 14).

**Fig. 3:**
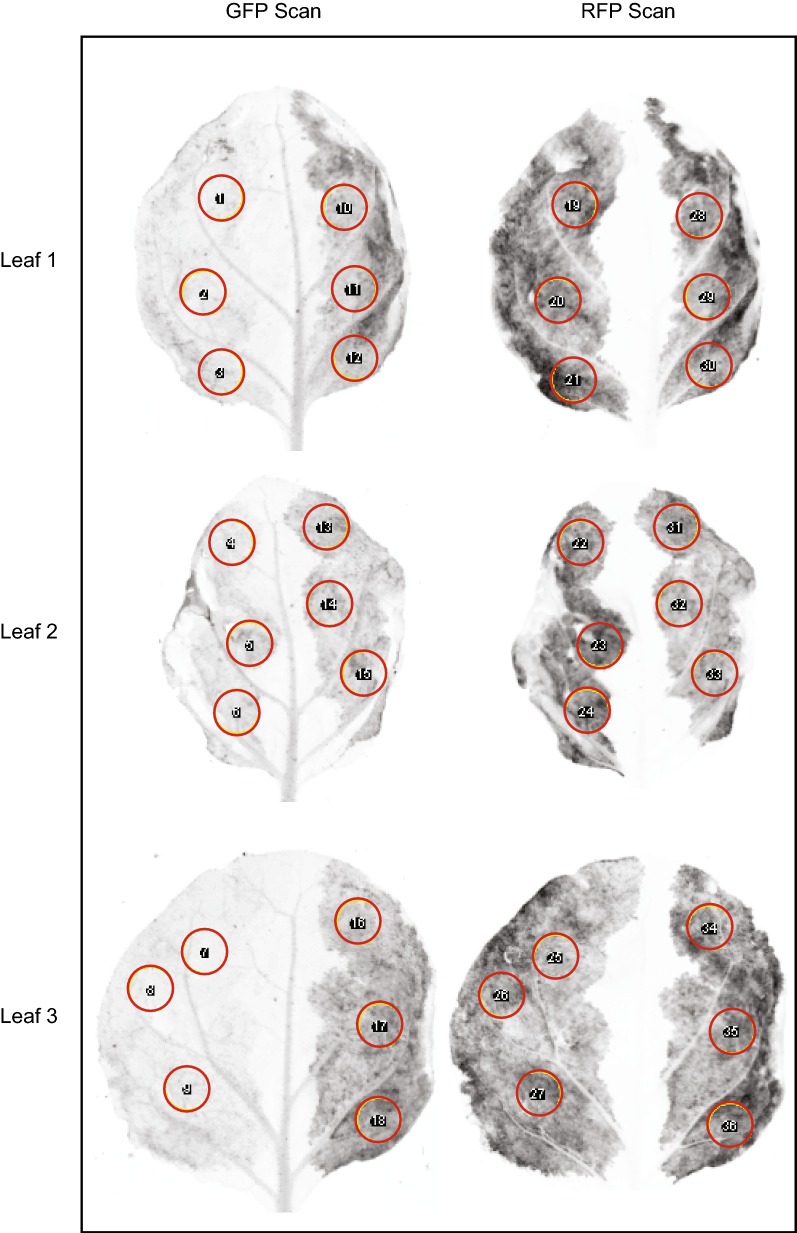
Demonstration of the tobacco leaf sgRNA efficiency assay. Each side of a tobacco leaf was infiltrated with one test construct. GFP and RFP fluorescence images were captured sequentially for the same leaf. The tests for a weak sgRNA (HCT_gRNA8) and a strong sgRNA (HCT_gRNA9) with three biological replicates are shown as Leafs 1, 2, and 3. Representative areas (red circles) on leaf were selected and measured for integrated intensity using ImageJ software. The ratio of GFP intensity vs. RFP intensity of the three representative areas was averaged for each biological replicate. The editing efficiency of each sgRNA was evaluated based on GFP:RFP ratio obtained from 5–7 biological replicates for each assay

Agrobacterium strains carrying the reporter construct and each of the test construct were grown separately to the stationary phase and mixed in equal amount for coinfiltration into half tobacco leaves (Fig. 3). Leaves for infiltration were carefully chosen from tobacco plants with similar growth status. Infiltrated plants were grown for 64 to 72 h to allow transgene expression and occurrence of CRISPR editing. Efficiency tests for sgRNAs of the same GOI were performed using the same batch of plants. Test results, i.e., GFP:RFP ratio for each test construct may vary using different batches of plants, but the trend of efficiency difference among sgRNAs remains the same.

We recorded a broad range of GFP:RFP ratios for the different *HCT* sgRNAs (Fig. 2b). Assays containing HCT_gRNA9 and 14 showed the highest GFP:RFP ratio, indicating the highest editing/frame-shift efficiency; HCT_gRNA3, 4, and 13 showed a medium–high GFP:RFP ratio, indicating medium editing efficiency; HCT_gRNA5, 6, 8, 10, 11, and 12 showed low editing efficiency with a slightly higher GFP:RFP ratio compared to the negative control sgRNA, which is a nontargeting sgRNA.

PCR amplification of the target DNA region followed by Sanger sequencing of the amplicon is frequently used to confirm genome editing events [33, 34]. Since a significant quantity of Agrobacteria remain in leaf tissues after transformation, we analyzed target regions on the *HCT* transcript rather than the genomic DNA sequence, as we expect that the DNA would be contaminated by the binary vector harbored by the Agrobacterium. Example sequencing results are shown for a low efficiency sgRNA (HCT_gRNA12) and a high efficiency sgRNA (HCT_gRNA14) (Fig. 2c). Mixed peaks in the sequencing chromatogram were detected at the expected sites of editing, i.e., 4 bp before the PAM sequence for each HCT_gRNA [9], indicating Cas9 cleavage and NHEJ -induced mutations at the target sites.

To confirm the results, immunoblot analysis of tobacco leaf protein extracts showed that in samples containing a nontargeting sgRNA or a sgRNA with low editing efficiency (represented by HCT_gRNA8), both Csy4 and cogGFP proteins were detected at a low level. In samples containing a sgRNA with high editing efficiency (represented by HCT_gRNA14), the cogGFP protein was very abundant while the Csy4 protein was barely detectable (Additional file 2). Together, these results support the successful execution of the experimental design (Fig. 1) and show that the occurrence of genome editing abolishes Csy4 protein accumulation which subsequently stops cogGFP mRNA degradation, leading to cogGFP mRNA translation and GFP protein accumulation.

### Comparison of the in vivo test results with bioinformatic predictions of sgRNA efficiency

Various algorithms for sgRNA efficiency prediction have been developed, and integrated into web-based tools for sgRNA evaluation [35–38]. We collected efficiency scores of the 14 HCT_gRNAs from two CRISPR websites: CRISPR-P2.0 (http://crispr.hzau.edu.cn/cgi-bin/CRISPR2/CRISPR, which is plant focused [35, 39]; and CHOPCHOP (http://chopchop.cbu.uib.no), which is more general and covers a wider array of species (Additional file 1) [36, 40]. For three sgRNAs (HCT_gRNA5, HCT_gRNA6 and HCT_gRNA13), the predicted efficiency from the two websites agreed with each other, as well as with the test results from the in vivo assay. However, no agreement on editing efficiency was found between the predicted results and the test results for the other sgRNAs. For example, HCT_gRNA3, HCT_gRNA4, HCT_gRNA9, and HCT_gRNA14 were predicted to have medium-to-high editing efficiency by CHOPCHOP, which was consistent with the experimental results of our assay, but they received a low score from CRISPR-P2.0. On the other hand, HCT_gRNA1, HCT_gRNA10, and HCT_gRNA11 showed low editing efficiency, agreeing with the CRISPR-P2.0 prediction but contradicting the prediction generated by CHOPCHOP (Additional file 1).

In one of the first studies which aimed to identify rules affecting sgRNA efficiency in plants, Liang et al. [41] retrieved sgRNA sequences from published plant CRISPR editing studies and analyzed their characteristics. They identified common features which include sgRNA G/C content between 30 and 80%, intact secondary structures, and various base-paring rules. The 14 HCT sgRNAs complied with almost all of these characteristics, with the exception of HCT_gRNA4 and HCT_gRNA7 (Additional file 1).

### Comparison of the transient assay system to stably transformed plants, using cell-specific editing

The best performer (HCT_gRNA14) and a weak performer (HCT_gRNA12), from the sgRNA efficiency screen were selected for further testing by generating stable Arabidopsis transformants. As described in “Introduction,” HCT is required in vessels, and reduced *HCT* expression in these cells has severe effects on plant growth and development. To specifically target *HCT* in fiber cells and minimize potential side effects of the engineering, we used a cell-type-specific promoter (*NST3*/*SND1*) to drive the expression of the gene editing construct. NST3/SND1 is a master regulator of secondary cell wall development in fiber cells [42, 43]. By utilizing the *NST3*/*SND1* promoter to drive *Cas9* expression, we aimed to generate mutation at the *HCT* loci only in the fiber cells, without affecting HCT activity in other, critical cell types (e.g., meristematic cells; photosynthetic cells and vessel forming cells). Editing efficiency is critical here, as ideally both HCT alleles in each cell of the target tissue should be mutagenized, to maximize the lignin reduction.

**Fig. 4:**
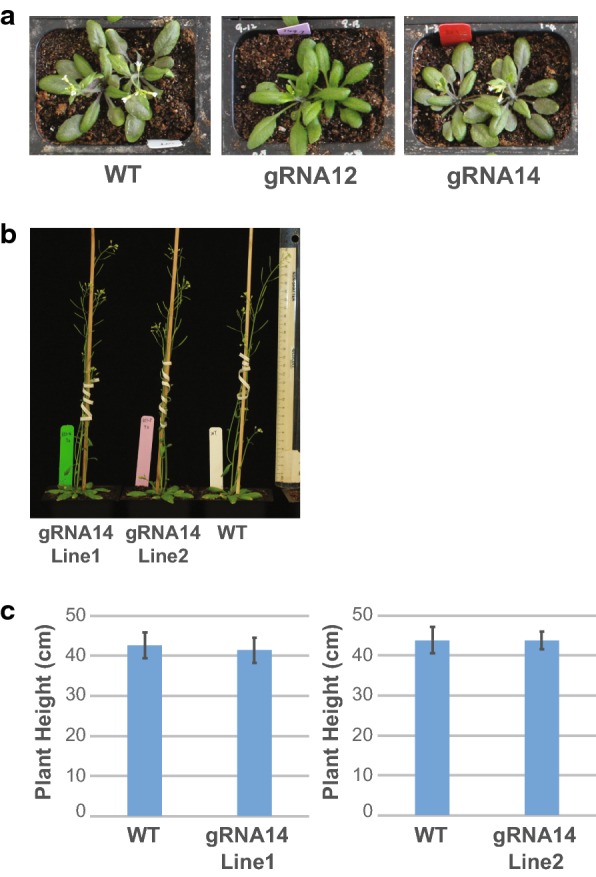
Phenotypic analysis of T1 and T2 pNST3::CAS9-pU6::HCT_gRNA14 plants. **a** Representative images of WT and T1 plants at 25 DPG. **b** Representative images of WT and T2 plants at 39 DPG. **c** Heights of WT and T2 plants. Data represent the mean ± SD of 6–7 biological replicates

The first transgenic generation (T1) plants containing pNST3::Cas9-pU6::HCT_gRNA12 or gRNA14 did not show visual phenotypic defects, as compared to WT plants (Fig. 4). One distinguishing feature of HCT-defective plants is the increase in H lignin units [26, 44, 45], which otherwise only form a small portion of the lignin composition of eudicots [46]. Pyrolysis–gas chromatography/mass spectrometry (Pyro–GC/MS) showed that the proportion of H-units was increased in two out of twelve independent transgenic plants carrying pNST3::Cas9-pU6::HCT_gRNA14 (Additional files 3 and 4). In contrast, none of the transgenic plants carrying pNST3::Cas9-pU6::HCT_gRNA12 (the low-efficiency sgRNA) showed a change in lignin composition compared to that of WT plants.

### Cell-specific editing was maintained in the T2 generation

**Fig. 5:**
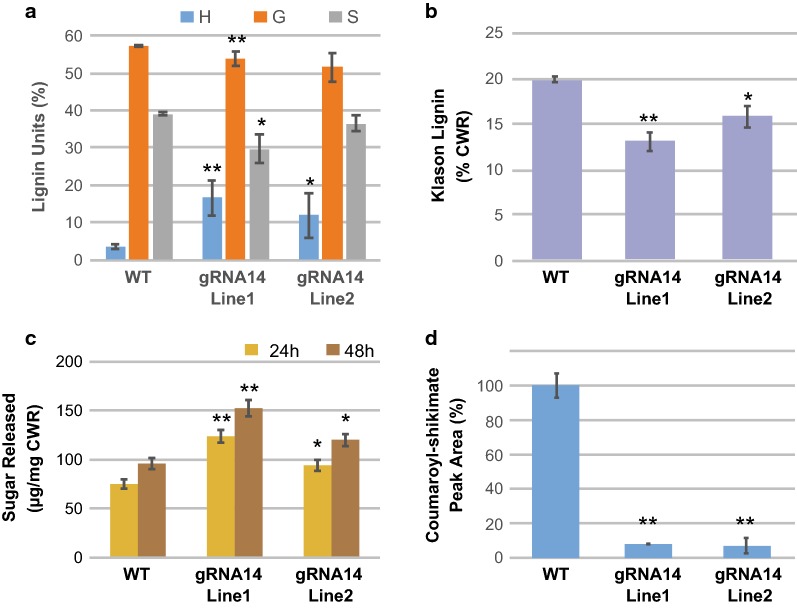
Biochemical phenotype of T2 pNST3::CAS9-pU6::HCT_gRNA14 plants. Senesced inflorescence stems from WT and transgenic plants (two independent lines) were analyzed for lignin monomer composition with Pyro–GC/MS analysis (**a**), lignin content with Klason method (**b**), and reducing sugars released after hot water pretreatment and enzymatic digestion (**c**). **d** Relative HCT activity was determined by measuring coumaroyl–shikimate formation in vitro. H, G, and S designate *p*-hydroxyphenyl, guaiacyl, and syringyl lignin units, respectively. Lignin monomer composition and saccharification analyses were performed with seven biological replicates; lignin content analysis was performed with 4–5 biological replicates; HCT activity was performed with 3–4 biological replicates. Mean value ± SD is shown. Asterisks indicate significant differences compared to WT using the unpaired Student’s *t*-test (*P < 0.05; **P < 0.005)

To determine the heritability and stability of the chimeric engineering approach, we analyzed HCT_gRNA14 Line1 and Line2 in their T2 generation. As with the T1 plants, the T2 transgenic plants did not show a visual difference in whole plant growth and plant height measurements (Fig. 4b, c), but did show a significant increase in H lignin units and a 20–30% reduction in total lignin compared to WT plants (Fig. 5; Additional file 4). Enzymatic saccharification after hot water pretreatment of the same material showed a 50% (Line 1) and 30% (Line 2) increase in reducing sugar release compared to WT plants (Fig. 5c). Supporting these data, analysis of HCT enzyme activity in the developing inflorescence stems revealed more than a 90% reduction in activity compared to WT plants (Fig. 5d).

### Testing the tissue specificity of the CRISPR/Cas9-mediated *HCT* editing

**Fig. 6:**
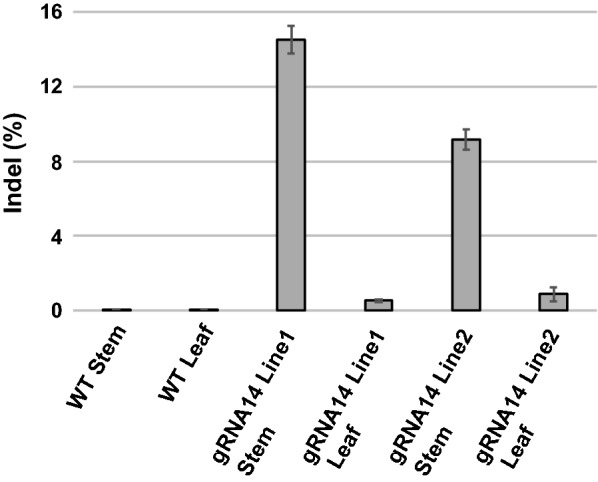
Detection of mutations at the HCT_gRNA14 target site in T2 pNST3::CAS9-pU6::HCT_gRNA14 plants. Genomic DNA was isolated from leaf and stem tissues, and genomic sequences spanning the HCT_gRNA14 target site were PCR amplified and analyzed by MiSeq sequencing. Mutation frequencies at 4 bp before the PAM sequence at the target site are shown. Values are mean ± SD of two biological replicates

To determine the changes to the *HCT* gene DNA sequence, genomic DNA was isolated from stem and leaf tissues of T2 transgenic plants carrying pNST3::Cas9-U6::HCT_gRNA14. The genomic region surrounding the HCT_gRNA14 target site was amplified and subjected to MiSeq deep sequencing. Indel rates at the target genomic region were compared among different plant lines (Fig. 6). In both stem and leaf tissues of WT plants, the indel rate at HCT_gRNA14 target site was approximately 0.05%, which is considered to be within the error for MiSeq analysis. For HCT_gRNA14 Line1 and Line2 plants, the indel rates of stem tissues were approximately 14% and 9%, respectively, while the indel rates of leaf tissues in the corresponding lines were approximately 0.5 and 0.9%.

To examine the spatial changes to lignin deposition, transverse sections of stems from HCT_gRNA14 plants were stained with phloroglucinol, which is commonly used for lignin in situ detection [47]. Lignified tissues of WT plants were stained purple, with vessel cell walls showing a slightly darker color compared to fiber cell walls (Additional file 5). A brighter and pinkish color was observed in fiber cells in both interfascicular tissues and vascular bundles of HCT_gRNA14 Line1 after phloroglucinol staining (Additional file 5) indicating a reduction of lignin content or changes in lignin composition [48]. The HCT_gRNA14 Line2 fiber cells showed a purple color with intermediate intensity to that of WT and HCT_gRNA14 Line1. The color change of vessel cells in vascular bundles was less distinguishable in both of the transgenic lines compared to WT. No irregular-xylem phenotype [49] or other morphological defects in stem anatomy were observed for transgenic plants compared to their WT counterparts.

An indel enrichment in the stem, together with fiber-specific reduction of phloroglucinol staining in stem sections of pNST3::Cas9-pU6::HCT_gRNA14 plants, supports the targeted editing of *HCT* in fiber tissues and demonstrates that editing of *HCT* was not passed through the germline.

### Testing the efficacy of the assay using germline editing of a nonessential gene

Reverse genetics is a common approach to assess gene function. In Arabidopsis research, for example, collections of publicly available T-DNA insertional mutants are extensively used. These mutant collections are not complete [50]. In addition, it is often important to make knockouts of multiple genes. However, if the genes are closely linked, this cannot be done via crossing two single mutants. Finally, sequenced mutant populations are only available for very few plant species. Therefore, efficient CRISPR editing is an important research tool.

As a second case study we chose a gene for which only a single mutant allele exists: *GONST2* (*Golgi-localized nucleotide sugar transporter 2*, At1g07290), which encodes a homolog of the GONST1 (Golgi-localized nucleotide sugar transporter 1, At2g13650) GDP-mannose transporter [51]. From the 108 candidate spacers selected with CRISPR-PLANT, we used manual selection to avoid targeting the closely related homologs in the *GONST* gene family [51, 52], and chose two to evaluate in the transient assay. GONST2_gRNA1 showed significantly higher editing efficiency compared to GONST2_gRNA2 for *GONST2* editing in the reporter construct (Additional file 6). Both sgRNAs were then used for editing endogenous *GONST2* in stable transgenic Arabidopsis plants. Since the goal of the experiment was to obtain a heritable mutation of *GONST2* gene in all cells, an Arabidopsis constitutive promoter, *UBQ10*, was used to drive Cas9 expression.

**Fig. 7:**
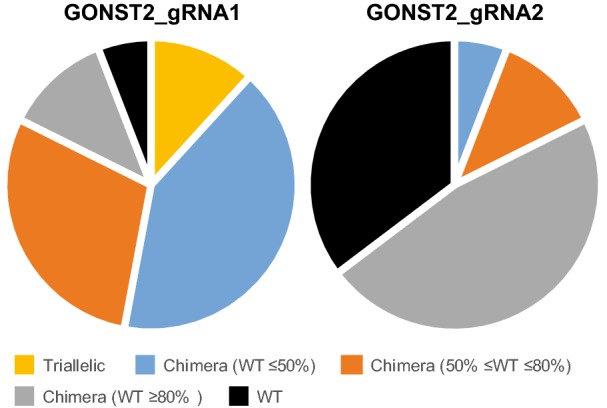
Zygosity of T1-edited *GONST2* plants. Following isolation of leaf DNA, genomic sequences spanning GONST2_gRNA1 or GONST2_gRNA2 target site were PCR amplified and analyzed by Sanger sequencing. Transgenic plants of each GONST2_gRNA line were classified based on mutation rate at the projected target site

T1 plants transformed with pUBQ10::Cas9-pU6::GONST2_gRNA1 or gRNA2 constructs were obtained, and 19 independent transgenic plants for each sgRNA were genotyped by Sanger sequencing of the target region (Additional file 7). The results were analyzed using TIDE (Tracking of Indels by Decomposition), a tool which identifies the type and frequency of small indels in the region close to the projected editing site [53]. The genotypes of the T1 plants and their relative proportion are summarized in Fig. 7. Supporting the data from the transient assay, GONST2_gRNA1 had much higher editing efficiency than gRNA2. Indeed, two out of nineteen T1 plants (~ 10%) were triallelic mutants. Overall more than half of GONST2_gRNA1 edited plants contained predominantly mutant alleles at the target site. In contrast, when GONST2_gRNA2 was used, one-third of the transgenic plants remained as WT at the target site.

For more in-depth analysis, amplicons from GONST2_gRNA1 plants #4 and #6 were cloned, and the individual clones were sequenced (Additional file 8). Sequencing confirmed absence of a WT allele. Plant #4 contained three indel types: single adenine insertion (73%), 46-bp deletion (23%), and 7-bp insertion (5%) at GONST2_gRNA1 target site. Plant #6 contained three indel types with approximately equal frequencies: single thymine insertion (39%), single cytosine insertion (28%), and a 19-bp insertion (33%).

## Discussion

The potential impact of sgRNA on CRISPR/Cas9 genome editing in plants has been recognized but not thoroughly studied [23–25, 41]. Using a transient tobacco expression system, Johnson and colleagues [23] first developed a sgRNA evaluation system in which a spacer sequence with a PAM motif was inserted in front of a frame-shifted luciferase reporter. Editing at the spacer sequence activated luciferase expression by shifting the luciferase sequence back to frame. Building on this system, our sgRNA efficiency assay is a positive readout system, which uses the expression level of GFP to measure genome editing efficiency. Our approach differs from Johnson’s study in that it uses either the open reading frame (as demonstrated here for *HCT*) or the entire genomic sequence (as demonstrated for *GONST2*) of the GOI included in the reporter construct, as opposed to just an isolated spacer sequence. This was achieved by employing a 2A peptide [28, 54] and Csy4 endonuclease [27] as an initial “off-switch” for GFP repression (Fig. 1). Given that inclusion of the open reading frame or the entire genomic sequence in the reporter construct in our assay system can better represent the context of the endogenous locus to be targeted, our assay should provide a more accurate estimation of the editing efficiency of the spacer sequences.

When the selected highly efficient HCT_gRNA14 was used to target the endogenous *HCT* gene in stable transgenic plants, an increase in H lignin units was detected in two out of twelve T1 CRISPR lines, which is consistent with changes observed in *hct* mutants in Arabidopsis [26], alfalfa [55, 56], and poplar [45]. We used a fiber-specific *NST3/SND1* promoter to drive *CAS9* expression and restrict editing to these cells. Presumably, the majority of cells in the stem, including parenchyma cells, xylem vessel cells, epidermal cells, etc. will retain the WT *HCT* allele, which is consistent with the 9 to 14% mutation rate detected for *HCT* gene in stems (Fig. 6). In addition, the minimal impacts on vessels and other cells in stems are evidenced by the normal morphology of the chimeric plant (Fig. 4). The 90% reduction in HCT enzyme activity in the stem (Fig. 5d), reduced phloroglucinol staining in fiber cells, and overall normal histology of stem transverse sections (Additional file 5) confirm a high mutation rate of the *HCT* loci in fiber cells, which represent living lignifying cells where *HCT* is highly expressed. In leaves, the 0.5–0.9% mutation rate of the *HCT* loci could be due to the presence of a small amount of fiber cells, or residual activity of the *NST3/SND1* promoter in leaf cells. In summary, this targeted approach allowed the generation of chimeric plants for the *HCT* loci, leading to reduced lignin and improved saccharification efficiency without impacting plant growth (Fig. 4). It also provides a method for exploring the effect of the loss of a gene in a specific cell type while keeping sequence integrity in germlines or meristems.

When Arabidopsis is transformed using the Agrobacterium-mediated floral dip method [57], ovules are the site of productive transformation and thus the transgene enters the zygote through the maternal DNA [58, 59]. CRISPR/Cas9 editing may happen in the female gametophyte or at any time during zygote development. During cell division, while the edited allele remains, the nonedited allele may yet be edited and acquire mutations of different types in different descendent cells. In most previous studies, including efforts to improve editing efficiency, plants lacking the WT allele of the target gene were only obtained in the T2 or T3 transgenic generation [33, 60–63]. Here, two out of the seventeen T1 lines with pUBQ10::Cas9-pU6::GONST2_gRNA1 were mutants of the *GONST2* gene at the target site with no WT allele detected. This suggests that the GONST2_gRNA1 enabled CRISPR/Cas9 editing during early embryo development, likely before the formation of shoot apical meristem. In support of the importance of an early editing onset, Wang et al. [64] obtained biallelic mutant of the target gene in the T1 generation by driving *Cas9* expression with an egg-specific promoter. It would be interesting to test whether combining an efficient sgRNA and the use of an egg-cell-specific promoter for Cas9 expression could achieve an even higher rate of mutagenesis in the T1 generation.

The sgRNAs showed an editing efficiency in the transient assay consistent with the efficiency in the stable transgenic plants. However, this did not necessarily correspond with predictions from the existing CRIPSR bioinformatics prediction tools, with only about a 50% match. The efficiency scores of different CRISPR tools, including the plant-focused CRIPSR-P2, were calculated based on algorithms developed with nonplant species [35, 36, 39, 40], which may not be completely applicable for plant species. Regardless of organism differences, only a moderate correlation between predicted and measured activity of sgRNAs, and modest concordance between the different algorithms have been observed [37]. Many factors have been suggested to affect sgRNA efficiency. With regard to the sequence, moderate GC content in the overall spacer sequence and the preference of GC in PAM proximal region were proposed as characteristics of efficient spacer sequences [15, 17, 19, 61]. In addition to a lower capacity for Cas9 binding, continuous T residues in the PAM proximal region were suggested to lower the sgRNA level by triggering transcriptional termination [16]. The crystal structure of the Cas9–sgRNA–DNA complex revealed the critical role of a T-shaped sgRNA:target DNA architecture in an active Cas9–sgRNA–DNA complex [20, 21]. Accordingly, base pairing within the spacer sequence or between a spacer and sgRNA backbone may interrupt the proper secondary structure of sgRNA and lower sgRNA efficiency [22]. In addition, the genomic context of the target site, designated by a spacer, also impacts the accessibility of the CRISPR/Cas9 editing machinery [16, 22]. These multiple factors may synergistically affect sgRNA efficiency. Therefore, it can be currently considered prudent to use a transient assay to determine sgRNA efficiency, as preselection may save substantial time and expense otherwise involved in transformation and posttransformation screening.

The current study demonstrates the use of CRISPR/Cas9 for tissue-specific gene targeting, to our knowledge, for the first time in plant studies. RNAi is a potent technique for systematic silencing of gene repression at the posttranscriptional level [65–67]. Tissue-specific targeting with RNAi was achieved for reproductive organs like seeds and floral organs [68, 69]; however, it is challenging for vegetative parts of plants due to the presence of conductive tissues and free diffusing properties of small RNA molecules, which lead to systemic silencing [70]. In contrast, the cell-to-cell movement of the Cas9 protein is unlikely, given that cell-to-cell shuttling is estimated to be limited to proteins < 40 kD [71]. In mammalian and yeast systems, the catalytically inactivated Cas9 protein, or dCas9, has been used for efficient gene repression either through direct dCas9 binding at the GOI or through conjugation to effector domains for the execution of a suppression effect [72–76], and this approach could provide an alternative strategy for tissue-specific engineering to the one we present here. In plants, gene repression has been demonstrated by means of a dCas9-repressor fusion to target promoter sequences [77, 78]. The repression efficiency was moderate, and it remains to be seen whether more efficient dCas9 gene repression can be achieved in plants by means of highly efficient sgRNAs, as in human cells [79]. On the other hand, if multiple genomic sites are to be targeted simultaneously in a synthetic biological circuit, binding-dependent repression of dCas9 is likely to be less potent compared to the Cas9 system.

## Conclusions

In summary, our study demonstrates the importance of the selection of highly efficient sgRNAs for CRISPR/Cas9-mediated genome editing in plants. A transient assay was developed for the evaluation of sgRNA efficiency in vivo. With a highly efficient sgRNA, the *HCT* gene was mutated in a tissue-specific manner, resulting in chimeric plants with improved extractability of cell wall sugars while maintaining normal growth. Presumably, high editing efficiency ensures the onset of editing in most of the target cells despite the limits in expression window and strength of a tissue-specific promoter such as the transcription factor promoter used here. For the unconstrained knockout of *GONST2*, highly efficient editing occurs at early stages of embryo development. Triallelic mutants in the *GONST2* gene were obtained in the T1 generation, which is one or two generations earlier than similar CRISPR/Cas9 mutagenesis studies in Arabidopsis [33, 60–63]. There are likely more applications for preselected efficient sgRNAs, for example, boosting the multiplexing efficiency in CRISPR/Cas9 editing by avoiding dilution effect of inefficient sgRNAs [22]. The selection method and application of efficient sgRNAs we developed here can be adapted to other plant species. Efficient genome editing may accelerate the speed of obtaining new genetic variants for both the fundamental study of gene functions and for breeding applications.

## Methods

### Vector construction

The gene fragments of pNOS::DsRED::tNOS, tG7-AmpR, pU6::HCT_gRNA1::tNOS, HCT::fsSTOP::2A::Cys4 and *CAS9* were chemically synthesized (GenScript, Piscataway, NJ, USA). The *DsRed* (GenBank ID: AB557594.1) and *AmpR* (GenBank ID: KX682236.1) sequences are publicly available. The *CAS9* sequence was *PcoCAS9* (GenBank ID: KF264451), as used in Li et al. [30]. The parts are listed in Additional file 9 and the sequence of the synthetic parts is listed in Additional file 10. Oligonucleotide primers used are listed in Additional file 11. All plasmids and sequence information are publicly available through the JBEI ICE registry (https://public-registry.jbei.org/login) [80]. Construct number, construct content, JBEI registry ID and building strategy of each construct are listed in Additional file 9.

Expression vector pTKan-p35S::attR1-GW-attR2 was acquired from JBEI ICE registry and used to sequentially build C50, C381, and C382 via In-Fusion cloning (Takara Bio USA, Inc., Mountain View, CA, USA). C382 was used as the backbone vector for constructing all test constructs via Gateway cloning (Thermo Fisher Scientific, Waltham, MA, USA). The first Entry clone containing a sgRNA was built by cloning the chemically synthesized pU6::HCT_gRNA1 fragment into pDONR/Zeo vector via a BP Gateway reaction. The resulting pDONR/Zeo-attL1-pU6::HCT_gRNA1-attL2 plasmid was used to build all the other Entry clones containing different sgRNAs. In brief, the pDONR/Zeo-attL1-pU6::HCT_gRNA1-attL2 clone was amplified as three PCR fragments with homologous sequences at each end and assembled through In-Fusion cloning as illustrated in Additional file 12. The initial reporter construct pTKan-p35S::HCT::2A::Cys4-pNOS::cogGFP (C267) was built based on pTKan-p35S::attR1-GW-attR2 vector via a Multisite Gateway reaction. To build the other reporter constructs, the *HCT* sequence was removed via AscI digestion and replaced with another GOI, e.g., *GONST2* in C378, via In-Fusion reaction.

### Bioinformatics

Potential spacer sequences were evaluated using multiple existing bioinformatics tools. First, a list of candidate spacer sequences for *HCT* and *GONST2* were identified using CRISPR-PLANT (http://www.genome.arizona.edu/crispr/CRISPRsearch.html) [31]. Fourteen spacer sequences located along the *HCT* open reading frame were manually selected from the initial list. For *GONST2* spacer selection, genomic sequences of GONST family members (GONST1, GONST2, GONST3/GGLT1,£ and GONST4/GFT1) were aligned. Two spacer sequences targeting *GONST2* but not the other GONST family members were selected for further testing. Next, CRISPR-P2.0 was used to generate spacer sequences for the same genes (http://crispr.hzau.edu.cn/cgi-bin/CRISPR2/CRISPR) [35, 39]. On the CRISPR Design page of CRISPR-P2.0, U6 was selected as the snoRNA promoter; Arabidopsis was selected as the target genome; gene ID was the input for Locus Tag; and default values were used for the other parameters. The generated list of candidate spacer sequences contained all of the previously identified 14 *HCT* and 2 *GONST2* spacer sequences. The output provides the total GC%, an on-target efficiency score, and secondary structure features of each spacer. Finally, CHOPCHOP (http://chopchop.cbu.uib.no/) [36, 40], was used with default parameters, and again, the selected 14 *HCT* spacer sequences and 2 *GONST2* spacer sequences were found in the list and their predicted efficiency scores were recorded. Secondary structures of sgRNAs were predicted with mfold (http://unafold.rna.albany.edu/?q=mfold/RNA-Folding-Form) [81]. The entire sequence including a spacer sequence and the following backbone scaffold was used as the input sequence. Default parameters were applied.

### Plant growth

Tobacco (*Nicotiana benthamiana* Domin) plants were grown in a growth chamber under 16/8 h and 26/24 °C day/night cycles. WT and transgenic *Arabidopsis thaliana* (L.) Heynh plants in the study were of *Col*-*0* ecotype. Plant growth conditions were 16/8 h day/night cycles, 120 µmol m^2^ s^1^, 23 °C and 60% humidity.

### Agrobacterium-mediated tobacco leaf transformation

Binary vectors based on the pTKan plasmid were transformed into *Agrobacterium tumefaciens* (Agrobacterium) strain GV3101 and tobacco leaf infiltration was performed as described previously [27]. Each coinfiltration mix contains three Agrobacterium strains: the strain carrying a reporter construct; the strain carrying a test construct with *CAS9* and an individual sgRNA to be tested; and the p19 strain for the suppression of the plant defense system [82, 83]. Each Agrobacterium strain was adjusted to a final OD_600_ = 0.3.

### Imaging and quantitation of fluorescent signals in tobacco leaves

Three days after infiltration, tobacco leaves were imaged using an Amersham Imager 600 (GE Healthcare Life Sciences). GFP and DsRed fluorescent signals were detected and imaged with the preset blue light settings (excitation 460 nm, Cy2:525BP20 filter) and green light settings (excitation 520 nm, Cy3:605BP40 filter), respectively. Exposure time is 30 s for GFP and 2 s for DsRed, which ensures that the florescent signals are detected but not saturated. Both GFP and DsRed signals were recorded as gray-scale images and quantitated using ROI Manager in ImageJ software (https://imagej.nih.gov/ij/index.html) [84]. In brief, three circular areas (0.72 × 0.72 cm^2^) were selected on representative regions of each biological replicate (Fig. 3). The fluorescent signal in the selected area was quantified using the integrated density (IntDen) with Measure function in ROI manager. The GFP:DsRed signal ratios of three representative regions were averaged for each biological replicate. Finally, the GFP:DsRed signal ratio of 5–7 biological replicates were represented as a box-plot (Microsoft Excel). An unpaired Student’s *t*-test was performed to compare the editing efficiency between the negative control replicates (NC) and sgRNA assay replicates.

### Protein analysis of sgRNA assay product in tobacco leaves

Tobacco leaves (150 mg fresh weight) were powdered and mixed with protein 300 µL extraction buffer (0.05 M Hepes–KOH pH 7, 0.4 M sucrose, 1 mM DTT, 5 mM MgCl_2_, 5 mM MnCl_2,_ 1 mM PMSF). Centrifugation (20,000×*g*, 5 min, 4 °C) was performed twice to remove cell debris, and the supernatant was retained. Protein concentration was determined using the Pierce BCA protein Assay Kit (Thermo Fisher Scientific). Approximately 50 μg protein of each sample was loaded and separated on an 8–16% SDS-PAGE polyacrylamide gradient gel. GFP and Csy4 were detected with a primary antibody against attB-tag [85] at 1:5000 dilution and a secondary antibody, Anti-Rabbit HRP (Thermo Fisher Scientific) at 1:20,000 dilution. After immunoblotting, the membrane was stained with Imperial Protein Stain (Thermo Fisher Scientific) to visualize protein loading.

### Sequencing analysis

For tobacco leaf samples, RNA isolation and reverse-transcription were performed as described in Liang et al. [27]. For *HCT*-targeted Arabidopsis plants, the second 15 cm from the base of main stems or pooled leaf samples were harvested at 36 days postgermination (DPG). For *GONST2*-targeted Arabidopsis plants, pooled leaf samples were harvested at 19 DPG. Genomic DNA was prepared using the CTAB method [86].

100 ng cDNA (for tobacco leaf samples) or DNA (for Arabidopsis samples) was used as the template for amplification of the target region using Phusion^®^ High-Fidelity DNA Polymerase (New England Biolabs, Ipswich, MA, USA). The amplification cycle number was 28. The amplicon was purified using DNA Clean & Concentrator (Zymo Research, Irvine, CA, USA) prior to submission for Sanger Sequencing or MiSeq sequencing by the DIVA-DNA Seq service at the Joint BioEnergy Institute (https://www.jbei.org/).

For Sanger sequencing, chromatograms of the sequencing results were analyzed for mutation rate with TIDE (https://tide.deskgen.com/) [53], using wild-type samples as the reference. For each sample, frequencies of wild-type allele and alleles with deletions were provided by TIDE. The frequency with which each of the four nucleotides is introduced immediately after the break site is calculated by multiplying total frequencies of insertional alleles at +1 position with percentage of each inserted nucleotide provided by TIDE.

The MiSeq sequencing results were analyzed with Genomics Integrative Viewer (IGV) version 2.3 [87, 88], using the wild-type sequence as the reference.

### Arabidopsis transformation

Agrobacterium strain GV3101 harboring the desired binary vectors was used to transform Arabidopsis plants with the floral dip method [57]. T1 and T2 transgenic plants were selected by Kanamycin resistance on solid Murashige and Skoog media supplemented with 1% (w/w) sucrose before transferring to soil.

### Lignin content and composition

Fully senesced, whole inflorescence stems were harvested and milled into fine powder. Lignin content was measured with Klason analysis [89] and lignin composition was measured with Pyro–GC/MS analysis [90]. Detailed experimental procedure follows description in Eudes et al. [91] except that pyrolysis of biomass was conducted as follows: 100 °C (10 min) to 300 °C at a rate of 10 °C/min; the final temperature was held for 2 min.

### Saccharification analysis

The same biomass samples used for the lignin content analysis above was also used for saccharification analysis. The biomass was subjected to hot water pretreatment and enzymatic digestion with Cellic CTec2 cellulase (Novozymes, Bagsværd, Denmark). The released reducing sugars were analyzed with 3,5-dinitrosalicylic acid assay. Experimental details were described in Eudes et al. [91].

### HCT activity analysis

The first 15 cm of the primary stem from Arabidopsis plants at 36 DPG were used for protein extraction. HCT activity assay was performed as described in Eudes et al. [92]. In brief, the in vitro assay used *p*-coumaroyl-CoA and shikimate as substrates and the reaction product was analyzed with HPLC–ESI-TOF MS as described in Eudes et al. [93].

#### Histochemical analysis of CRISPR *HCT* editing plants

Transverse sections were made from the base of the main stem of WT and transgenic plants at 39 days postgermination. Sectioning and phloroglucinol staining were processed as described in Mitra and Loqué [47].

### Sequence IDs

The promoters and coding sequences used in the gene constructs relate to the following IDs: GONST1, At2g13650; GONST2, At1g07290; HCT, At5g48930; NST3/SND1, At1g32770; UBQ10, At4g05320.

## Additional files

**Additional file 1.** Comparison of predicted sgRNA efficiency to actual sgRNA efficiency shown in tobacco transient assay.

**Additional file 2.** Immunoblot analysis of GFP and Csy4 protein expression in the sgRNA efficiency assays.

**Additional file 3.** Lignin composition of T1 plants with fiber cell-specific *HCT* editing.

**Additional file 4.** Characteristics and relative molar abundances (%) of the compounds released after Pyro-GC/MS of extractive-free senesced mature stems from WT, pNST3::CAS9-pU6::HCT_gRNA12 (gRNA12), and pNST3::CAS9-pU6::HCT_gRNA14 (gRNA14) plants.

**Additional file 5.** Phloroglucinol staining of stem transverse sections from T2 pNST3::CAS9-pU6::HCT_gRNA14 plants.

**Additional file 6.** Editing efficiency of the two screened GONST2_gRNAs.

**Additional file 7.** Zygosity of the T1 generation of *GONST2* targeted Arabidopsis plants.

**Additional file 8.** Mutation analysis of T1 transgenic plants of pUBQ10::CAS9-pU6::GONST2_gRNA1.

**Additional file 9.** Expression clones and their building parts.

**Additional file 10.** Sequence of the synthetic parts.

**Additional file 11.** Primer list.

**Additional file 12.** A map demonstrating In-Fusion cloning of Entry Clones containing individual sgRNAs.

## Data Availability

All data generated or analyzed during this study are included in this published article and its supplementary information files, or are available from the corresponding authors on reasonable request. All constructs and seeds are available via the JBEI registry (https://registry.jbei.org/).
