# Supplementary material for: A screening method to identify efficient sgRNAs in Arabidopsis, used in conjunction with cell-specific lignin reduction
Source: Biotechnol Biofuels. 2019 May 23;12:130. doi: 10.1186/s13068-019-1467-y (PMC6532251; doi:10.1186/s13068-019-1467-y)
Supplement: Supplementary file 1 — Additional file 1. Comparison of predicted sgRNA efficiency to actual sgRNA efficiency shown in tobacco transient assay. [file 13068_2019_1467_MOESM1_ESM.pdf]

Additional File 1. Comparison of predicted sgRNA efficiency to actual sgRNA efficiency shown in tobacco transient assay.

Name	Sequence			CRISPR-PLANT[1]	Plant sgRNA Characteristics[2]			CRISPR-P2.0[3]	CHOPCHOP[4]	Efficiency Assay[5]
	Protospacer	PAM	Strand	Highly specific: Class 0.0 or 1.0	Total GC %[6] [30, 80]: Efficient	Secondary structure features[6]: CBP≤7; TBP≤12;IBP≤6: Efficient	Secondary structure[7] integrity: Repeat-anti-Repeat, Stem Loop 2 and Stem Loop 3 are essential; Stem Loop 1 is optional	Efficiency Score[0, 0.2] : Inefficient; [0.2, 0.5]: Intermediate; [0.5, 1]: Efficient	Efficiency Score	Relative GFP:RFP Ratio
HCT_gRNA1	GTCGCTTGAAGAGAGACGATGA	TGG	-	1.0	50%	3 CBP;9 TBP;0 IBP	All Structures Detected	0.08	0.51	N.D.
HCT_gRNA2	GTCTACTTCTACAGACCCAC	CGG	-	1.0	50%	5 CBP;13 TBP;0 IBP	Stem Loop 1 Missing	0.67	0.51	N.D.
HCT_gRNA3	GGTATGTGCACCGCGGACAA	GGG	+	0.0	60%	6 CBP;10 TBP;3 IBP	Repeat-anti-Repeat Altered	0	0.64	Medium
HCT_gRNA4	GCTGTGGCGGGTCCCTAGCT	CGG	+	0.0	70%	8 CBP;14 TBP;0 IBP	Stem Loop 1 Missing	0.13	0.51	Medium
HCT_gRNA5	GACAGTGTTCCCATCCTCCT	TGG	+	1.0	55%	3 CBP;5 TBP;2 IBP	Stem Loop 1 Missing	0.07	0.38	Low
HCT_gRNA6	GAGGTCAGCTCTTGACTACC	TGG	-	1.0	55%	7 CBP;10 TBP;3 IBP	Stem Loop 1 Missing	0.05	0.34	Low
HCT_gRNA7	GATAAGCTGCCATCATTAGT	AGG	+	1.0	45%	11 CBP;12 TBP;0 IBP	Stem Loop 1 Missing	0.48	0.49	N.D.
HCT_gRNA8	GATATTCACTGCAACACCAT	TGG	-	1.0	40%	4 CBP;11 TBP;0 IBP	Stem Loop 1 Missing	0.28	0.51	Low
HCT_gRNA9	GATCAGTGGGAAAGGCGCGA	GGG	-	1.0	60%	3 CBP;10 TBP;0 IBP	Stem Loop 1 Missing	0	0.67	High
HCT_gRNA10	GATCTGTCAGCCCTTGTCCG	CGG	-	1.0	60%	3 CBP;10 TBP;0 IBP	Stem Loop 1 Missing	0	0.59	Low
HCT_gRNA11	GATGAAAAGCAGGCTGTGGC	GGG	-	1.0	55%	5 CBP;11 TBP;0 IBP	Stem Loop 1 Missing	0.14	0.48	Low
HCT_gRNA12	GCTCATACGAGATGTTGGCA	GGG	-	1.0	50%	4 CBP;10 TBP;0 IBP	Stem Loop 1 Missing	0.51	0.58	Low
HCT_gRNA13	GTATGGATGATAACTATCTG	AGG	-	1.0	35%	7 CBP;10 TBP;0 IBP	Stem Loop 1 Missing	0.55	0.57	Medium
HCT_gRNA14	GTGTTCAGATTGGAGGGCAA	TGG	+	1.0	50%	4 CBP;8 TBP;0 IBP	Stem Loop 1 Missing	0.24	0.55	High
GONST2_gRNA1	GAGATAACAGGCGTGACCAC	GGG	-	0.0	55%	6 CBP;12 TBP;0 IBP	Stem Loop 1 Missing	0.33	0.58	High
GONST2_gRNA2	GTTTGGTGGGTTCATTAAACA	AGG	-	1.0	35%	6 CBP;11 TBP;0 IBP	All Structures Detected	0.08	0.57	Low

[1]sgRNA specificity score from CRISPR-PLANT (http://www.genome.arizona.edu/crispr/CRISPRsearch.html).

[2]Criteria used for prediction of sgRNA efficiency in plants inlude total GC%, secondary structure features and integritys (Liang et al. 2016). Characters predicted for inefficient sgRNA was shown in red.

[3]sgRNA efficiency score and criteria from CRISPR-P2.0 (http://crispr.hzau.edu.cn/cgi-bin/CRISPR2/CRISPR). Agreement or disagreement of the prediction with tobacco assay results was shown in blue and red color respectively.

[4]sgRNA efficiency score and criteria from CHOPCHOP (http://chopchop.cbu.uib.no/). Agreement or disagreement of the prediction with tobacco assay results was shown in blue and red color respectively.

[5]sgRNA efficiency evaluation using the tobacco assay in this study (Fig. 2). N.D. : no activity was detected with statistical significance.

[6]Numbers for total GC% and secondary structure predicted by CRISPR-P2.0 (http://crispr.hzau.edu.cn/cgi-bin/CRISPR2/CRISPR). TBP, total base pairs; CBP, consecutive base pairs; IBP, internal base pairs; SL, stem loop.

[7]Secondary structure of sgRNA predicted by mFold (http://unafold.rna.albany.edu/?q=mfold/RNA-Folding-Form).
