# Supplementary material for: A screening method to identify efficient sgRNAs in Arabidopsis, used in conjunction with cell-specific lignin reduction
Source: Biotechnol Biofuels. 2019 May 23;12:130. doi: 10.1186/s13068-019-1467-y (PMC6532251; doi:10.1186/s13068-019-1467-y)
Supplement: Supplementary file 2 — Additional file 2. Immunoblot analysis of GFP and Csy4 protein expression in the sgRNA efficiency assays. [file 13068_2019_1467_MOESM2_ESM.pdf]

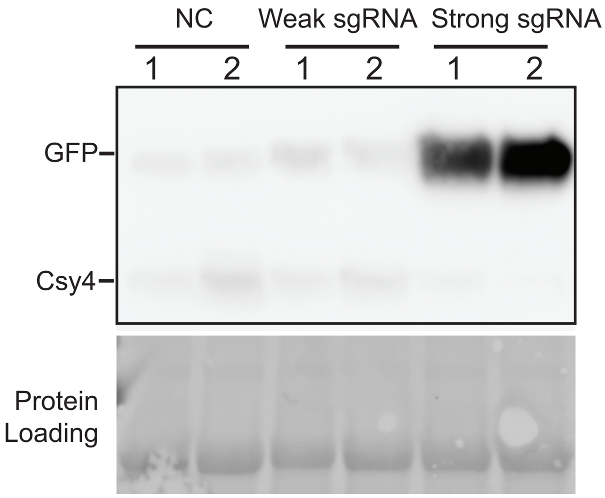

Additional File 2. Immunoblot analysis of GFP and Csy4 protein expression in the sgRNA efficiency assays. Protein samples were prepared from tobacco leaves infiltrated with an assay for either a negative control (NC), a weak sgRNA (HCT_gRNA8) or strong sgRNA(HCT_gRNA14). Both GFP and Csy4 proteins were detected using an attB2-tag antibody (upper panel). Equal loading of samples is shown using total protein staining (lower panel). Two biological replicates are shown.
