# Supplementary material for: A screening method to identify efficient sgRNAs in Arabidopsis, used in conjunction with cell-specific lignin reduction
Source: Biotechnol Biofuels. 2019 May 23;12:130. doi: 10.1186/s13068-019-1467-y (PMC6532251; doi:10.1186/s13068-019-1467-y)
Supplement: Supplementary file 3 — Additional file 3. Lignin composition of T1 plants with fiber cell-specific HCT editing. [file 13068_2019_1467_MOESM3_ESM.pdf]

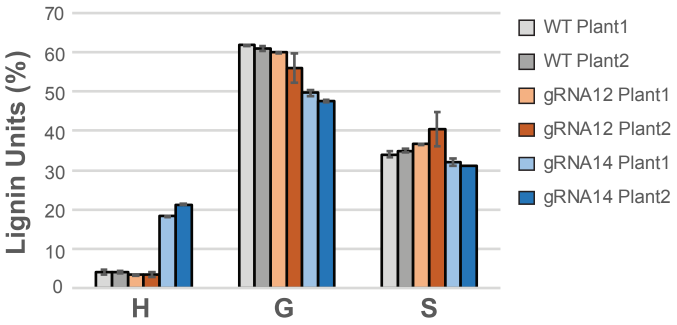

Additional File 3. Lignin composition of T1 plants with fiber cell-specific ***HCT*** editing. Based on the results from the transient assay, HCT_gRNA12 and HCT_gRNA14 represent a less efficient sgRNA and a highly efficient sgRNA respectively. H, G, and S designate p-hydroxy-phenyl, guaiacyl, and syringyl lignin units respectively. Results of two independent transgenic plants of each genotype are shown. Values were means ± SD of two technical replicates.
