# Supplementary material for: A screening method to identify efficient sgRNAs in Arabidopsis, used in conjunction with cell-specific lignin reduction
Source: Biotechnol Biofuels. 2019 May 23;12:130. doi: 10.1186/s13068-019-1467-y (PMC6532251; doi:10.1186/s13068-019-1467-y)
Supplement: Supplementary file 4 — Additional file 4. Characteristics and relative molar abundances (%) of the compounds released after Pyro-GC/MS of extractive-free senesced mature stems from WT, pNST3::CAS9-pU6::HCT_gRNA12 (gRNA12), and pNST3::CAS9-pU6::HCT_gRNA14 (gRNA14) plants. [file 13068_2019_1467_MOESM4_ESM.pdf]

Additional File 4. Characteristics and relative molar abundances (%) of the compounds released after pyro-GC/MS of extractive-free senesced mature stems from WT, pNST3::CAS9-pU6::HCT_gRNA12 (gRNA12), and pNST3::CAS9-pU6::HCT_gRNA14 (gRNA14) plants.

i) Analysis of T1 plants of pNST3::CAS9-pU6::HCT_gRNA12 line and pNST3::CAS9-pU6::HCT_gRNA14 line. Standard Deviation (STD) was calculated from two technical replicates

**Compound Name**	**Origin**	**Formula**	**MW**	**Main Mass Fragments**	**Retention Time (min)**	**WT-1 (%)**	**STD (%)**	**WT-2 (%)**	**STD (%)**	**gRNA12-1 (%)**	**STD (%)**	**gRNA12-2 (%)**	**STD (%)**	**gRNA14-1 (%)**	**STD (%)**	**gRNA14-2 (%)**	**STD (%)**
phenol	H	C6H6O	94	65, 66, 94	3.55	1.7	0.2	1.8	0.3	1.4	0.1	1.4	0.2	8.4	0.2	10.2	0.1
2,5-dimethylphenol	H	C8H10O	122	77, 79 108	5.26	1.4	0.3	1.3	0.0	1.2	0.1	1.1	0.3	6.0	0.1	6.6	0.1
2-methoxyphenol	G	C7H8O2	124	81, 109, 124	5.69	9.7	0.8	9.6	0.2	8.8	0.1	8.4	0.6	7.5	0.3	7.7	0.1
3-methylphenol	H	C7H8O	108	77, 107, 122	8.51	0.9	0.1	0.9	0.0	0.9	0.0	1.0	0.2	3.9	0.1	4.4	0.0
2-methoxy-5-methylphenol	G	C8H10O2	138	95, 123, 138	9.56	3.6	0.1	3.3	0.0	3.5	0.0	3.2	0.4	3.0	0.0	2.5	0.1
4-ethyl-2-methoxyphenol	G	C9H12O2	152	122, 137, 152	12.86	4.5	0.1	4.3	0.3	3.9	0.1	3.8	0.3	4.1	0.1	4.0	0.1
4-ethenyl-2-methoxyphenol	G	C9H10O2	150	107, 135, 150	13.9	21.3	0.1	21.1	0.2	21.1	0.5	19.5	1.1	17.3	0.4	16.6	0.2
phenol 2,6-dimethoxy	S	C8H10O3	154	65, 93, 139, 154	14.76	7.9	0.0	8.3	0.1	8.3	0.1	8.0	0.1	7.8	0.1	7.6	0.5
vanillin	G	C8H8O3	152	151, 152, 164	15.8	1.4	0.1	1.5	0.1	1.3	0.0	1.2	0.1	1.2	0.1	1.1	0.2
4-Methyl-2,6-dimethoxyphenol	G	C9H12O2	168	131, 149, 164	15.87	1.7	0.0	1.8	0.1	1.7	0.0	1.5	0.0	1.0	0.0	0.9	0.1
2-methoxy-4-(1-propenyl)phenol	G	C10H12O2	164	125, 153, 168	16.55	2.5	0.1	2.4	0.1	2.5	0.1	2.6	0.2	2.3	0.2	2.4	0.1
4-Methyl-2,6-dimethoxyphenol	G	C9H12O2	168	131, 149, 164	16.69	13.5	0.3	13.0	0.2	13.2	0.3	12.0	0.7	9.4	0.0	8.9	0.3
4-Ethyl-2,6-dimethoxyphenol	S	C10H14O3	182	167, 182	17.8	2.7	0.2	2.6	0.1	2.9	0.2	2.9	0.5	2.9	0.1	3.0	0.2
4-Hydroxy-3-methoxyphenyl acetone	G	C10H12O3	180	122, 137, 180	17.9	3.7	0.1	3.9	0.6	4.0	0.0	3.8	0.3	3.6	0.0	3.6	0.3
4-Hydroxy-3,5-dimethoxystyrene	S	C10H12O3	180	91, 137, 165, 180	18.39	13.9	0.4	14.0	0.4	14.7	0.1	14.3	0.8	12.6	1.0	12.1	0.0
4-allyl-2,6-dimetoxyphenol	S	C11H14O3	194	167, 179, 194	18.86	1.3	0.1	1.3	0.3	1.5	0.2	6.2	6.6	1.1	0.0	1.0	0.0
4-(2-Propenyl)-2,6-dimethoxyphenol	S	C11H12O3	192	91, 119, 151, 194	19.5	0.9	0.1	1.1	0.2	1.1	0.1	1.1	0.0	1.0	0.1	0.8	0.1
trans-4-propenyl-2,6-dimetoxyphenol	S	C11H14O3	194	167, 179, 194	20.18	7.3	0.0	7.7	0.1	8.1	0.0	8.0	0.8	6.7	0.0	6.5	0.1
**H-unit (%)**						**4.1**	0.7	**4.1**	0.3	**3.5**	0.0	**3.5**	0.7	**18.3**	0.2	**21.3**	0.0
**G-unit (%)**						**61.9**	0.1	**60.9**	0.7	**59.9**	0.0	**56.0**	3.7	**49.7**	0.7	**47.7**	0.3
**S-unit (%)**						**34.0**	0.8	**34.9**	0.4	**36.6**	0.0	**40.5**	4.4	**32.1**	0.9	**31.0**	0.3

ii) Analysis of T2 plants of two lines of pNST3::CAS9-pU6::HCT_gRNA14. Standard Deviation (STD) was calculated from seven biological replicates for the transgenic lines and four biological replicates for WT plant.

**Compound name**	**Origin**	**Formula**	**MW**	**Main mass fragments**	**Retention Time(min) (min)**	**WT (%)**	**STD (%)**	**gRNA14 Line1 (%)**	**STD (%)**	**gRNA14 Line2 (%)**	**STD (%)**
phenol	H	C6H6O	94	65, 66, 94	3.55	1.8	0.5	10.3	3.2	6.5	3.4
2,5-dimethylphenol	H	C8H10O	122	77, 79 108	5.26	1.3	0.2	3.4	1.1	3.9	1.8
2-methoxyphenol	G	C7H8O2	124	81, 109, 124	5.69	6.6	0.5	15.3	2.2	6.7	0.6
3-methylphenol	H	C7H8O	108	77, 107, 122	8.51	0.4	0.1	2.8	0.7	1.5	0.7
2-methoxy-5-methylphenol	G	C8H10O2	138	95, 123, 138	9.56	2.4	0.4	2.0	0.4	1.9	0.1
4-ethyl-2-methoxyphenol	G	C9H12O2	152	122, 137, 152	12.86	3.0	0.4	2.5	0.4	3.8	0.2
4-ethenyl-2-methoxyphenol	G	C9H10O2	150	107, 135, 150	13.9	19.8	0.8	13.6	1.0	17.6	2.8
phenol 2,6-dimethoxy	S	C8H10O3	154	65, 93, 139, 154	14.76	6.8	0.7	6.3	1.0	7.7	0.9
vanillin	G	C8H8O3	152	151, 152, 164	15.8	2.6	0.6	5.5	0.6	1.8	0.4
4-Methyl-2,6-dimethoxyphenol	G	C9H12O2	168	131, 149, 164	15.87	2.3	0.3	2.5	0.6	2.3	0.2
2-methoxy-4-(1-propenyl)phenol	G	C10H12O2	164	125, 153, 168	16.55	3.4	0.4	1.8	0.2	2.9	0.3
4-Methyl-2,6-dimethoxyphenol	G	C9H12O2	168	131, 149, 164	16.69	12.9	0.6	8.5	0.9	11.0	1.2
4-Ethyl-2,6-dimethoxyphenol	S	C10H14O3	182	167, 182	17.8	3.0	0.4	2.5	0.7	3.4	0.3
4-Hydroxy-3-methoxyphenyl acetone	G	C10H12O3	180	122, 137, 180	17.9	4.3	0.4	2.0	0.4	3.6	0.3
4-Hydroxy-3,5-dimethoxystyrene	S	C10H12O3	180	91, 137, 165, 180	18.39	15.8	1.0	11.0	1.5	13.8	1.8
4-allyl-2,6-dimetoxyphenol	S	C11H14O3	194	167, 179, 194	18.86	2.6	0.0	1.6	0.2	1.6	0.4
4-(2-Propenyl)-2,6-dimethoxyphenol	S	C11H12O3	192	91, 119, 151, 194	19.5	2.0	0.3	1.6	0.2	2.0	0.4
trans-4-propenyl-2,6-dimetoxyphenol	S	C11H14O3	194	167, 179, 194	20.18	9.0	0.8	6.5	1.0	8.1	0.9
**H-unit (%)**						**3.5**	0.7	**16.5**	4.8	**11.8**	6.0
**G-unit (%)**						**57.4**	0.2	**53.9**	1.8	**51.7**	3.9
**S-unit (%)**						**39.1**	0.6	**29.6**	3.9	**36.5**	2.3
