# Supplementary material for: A screening method to identify efficient sgRNAs in Arabidopsis, used in conjunction with cell-specific lignin reduction
Source: Biotechnol Biofuels. 2019 May 23;12:130. doi: 10.1186/s13068-019-1467-y (PMC6532251; doi:10.1186/s13068-019-1467-y)
Supplement: Supplementary file 5 — Additional file 5. Phloroglucinol staining of stem transverse sections from T2 pNST3::CAS9-pU6::HCT_gRNA14 plants. [file 13068_2019_1467_MOESM5_ESM.pdf]

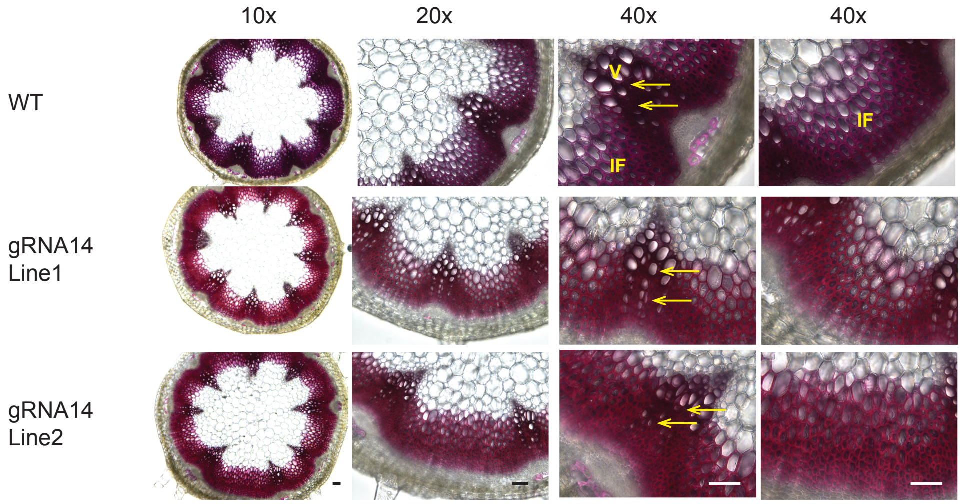

Additional File 5. Phloroglucinol staining of stem transverse sections from T2 pNST3::-CAS9-pU6::HCT_gRNA14 plants. Transverse sections were made from the base of the main stem of WT and transgenic plants at 39 DPG. V: vessel cells; IF: interfascicular fiber cells. Arrows indicate fiber cells surrounding the vessel cells in the xylem. Scale bar: 50 µm.
