# Supplementary material for: A screening method to identify efficient sgRNAs in Arabidopsis, used in conjunction with cell-specific lignin reduction
Source: Biotechnol Biofuels. 2019 May 23;12:130. doi: 10.1186/s13068-019-1467-y (PMC6532251; doi:10.1186/s13068-019-1467-y)
Supplement: Supplementary file 6 — Additional file 6. Editing efficiency of the two screened GONST2_gRNAs. [file 13068_2019_1467_MOESM6_ESM.pdf]

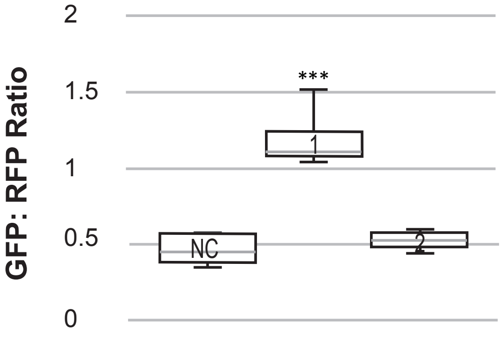

Additional File 6. Editing efficiency of the two screened GONST2_gRNAs. The relative efficiency of individual GONST2_gRNA is expressed as GFP:RFP ratios. NC, negative control. Data represent the mean + SD of 5-7 biological replicates. For details of the individual sgRNAs, see Additional File1. Asterisks indicate significant differences compared to NC using the unpaired Student's *t*-test (***$P < 0.001$).
