# Supplementary material for: A screening method to identify efficient sgRNAs in Arabidopsis, used in conjunction with cell-specific lignin reduction
Source: Biotechnol Biofuels. 2019 May 23;12:130. doi: 10.1186/s13068-019-1467-y (PMC6532251; doi:10.1186/s13068-019-1467-y)
Supplement: Supplementary file 7 — Additional file 7. Zygosity of the T1 generation of GONST2 targeted Arabidopsis plants. [file 13068_2019_1467_MOESM7_ESM.pdf]

Additional File 7. Zygosity of the T1 generation of *GONST2* targeted Arabidopsis plants. Sanger sequencing results of amplicons of the GONST2_gRNA targeted regions were analyzed with the TIDE (https://tide.deskgen.com/) bioinformatics tool (Brinkman et al. 2014).

i) Analysis on pUBQ10::CAS9-pU6::GONST2_gRNA1 plants

T1 Plants	Total Indel Efficiency (%)	Fit of Goodness R^2	Allele Genotypes and Their Percentage						Classification
			1st	2nd	3rd	4th	5th	6th	
Plant1	71	0.95	A Ins, 44.1%	WT, 24%	T Ins, 11.4%	C Ins, 6.7%	Del, 5.1%	-	Chi (WT ≤50%)
Plant3	11.3	0.95	WT, 85.7%	T Ins, 7.3%	A Ins, 2.0%	G Ins, 0.1%	-	-	Chi (WT >80%)
Plant4	92	0.93	A Ins, 77.7%	**Undefined**	C Ins, 2.2%	T Ins, 0.2%	-	-	Chi Mutant
Plant5	49.1	0.96	WT, 46.8%	T Ins, 45.5%	C Ins, 2.4%	A Ins, 0.3%	G Ins, 0.1%	-	Chi (WT ≤50%)
Plant6	65.3	**0.69**	**Not estimated**	-	-	-	-	-	Chi Mutant
Plant7	66.7	0.95	T Ins, 35.4%	WT, 28.2%	Del, 15.6%	A Ins, 14.0%	-	-	Chi (WT ≤50%)
Plant8	19	0.96	WT, 77%	T Ins, 5.3%	G Ins, 4.6%	A Ins, 3.6%	Del, 3.5%	-	Chi (50% <WT ≤80%)
Plant9	13.8	0.98	WT, 84%	T Ins, 5.9%	A Ins, 3.3%	G Ins, 1.4%	-	-	Chi (WT >80%)
Plant10	35.9	0.96	WT, 60.2%	G Ins, 16.2%	C Ins, 8.5%	T Ins, 6.5%	A Ins, 3.1%	-	Chi (50% <WT ≤80%)
Plant11	24.1	0.96	WT, 71.8%	T Ins, 12.6%	A Ins, 4.9%	Del, 3.9%	G Ins, 1.2%	-	Chi (50% <WT ≤80%)
Plant12	76.4	0.97	C Ins, 37.5%	T Ins, 36.8%	WT, 21%	G Ins, 0.9%	A Ins, 0.2%	-	Chi (WT ≤50%)
Plant13	84.9	0.96	C Ins, 38.7%	T Ins, 38.0%	WT, 11.6%	Del, 4.4%	G Ins, 0.9%	A Ins, 0.2%	Chi (WT ≤50%)
Plant14	42.5	0.97	WT, 54.1%	T Ins, 13.2%	A Ins, 9.7%	C Ins, 7.7%	Del, 5.9%	G Ins, 4.9%	Chi (50% <WT ≤80%)
Plant16	90.8	0.99	T Ins, 87.3%	WT, 8%	C Ins, 1.9%	A Ins, 1.1%	-	-	Chi (WT ≤50%)
Plant17	77.4	0.98	Del, 50.1%	WT, 20.2%	T Ins, 11.6%	A Ins, 8.7%	G Ins, 4.3%	C Ins, 2.4%	Chi (WT ≤50%)
Plant18	1.5	0.99	WT, 97.3%	-	-	-	-	-	WT
Plant19	39.9	0.97	WT, 57.3%	T Ins, 16.8%	A Ins, 13.7%	C Ins, 6.9%	Del, 1.6%	G Ins, 0.6%	Chi (50% <WT ≤80%)

ii) Analysis on pUBQ10::CAS9-pU6::GONST2_gRNA2 plants

T1 Plants	Total Indel Efficiency (%)	Fit of Goodness R^2	Allele Genotypes and Their Percentage						Classification
			1st	2nd	3rd	4th	5th	6th	
Plant1	4	0.97	WT, 93.3%	-	-	-	-	-	WT
Plant3	1.2	0.99	WT, 97.9%	-	-	-	-	-	WT
Plant4	35.3	0.98	WT, 62.4%	A Ins, 33.8%	T Ins, 1.0%	-	-	-	Chi (50% <WT ≤80%)
Plant5	3	0.99	WT, 95.9%	T Ins, 1.4%	G Ins, 1.0%	-	-	-	Chi (WT >80%)
Plant6	5.6	0.98	WT, 92.4%	A Ins, 4.1%	T Ins, 0.7%	G Ins, 0.3%	-	-	Chi (WT >80%)
Plant7	2.4	0.99	WT, 96.7%	A Ins, 2.2%	T Ins, 0.1%	-	-	-	Chi (WT >80%)
Plant8	15.4	0.99	WT, 83.9%	A Ins, 12.0%	T Ins, 0.6%	G Ins, 0.5%	-	-	Chi (WT >80%)
Plant10	0.3	0.99	WT, 98.9%	-	-	-	-	-	WT
Plant11	10.2	0.99	WT, 88.5%	A Ins, 6.8%	T Ins, 0.3%	G Ins, 0.2%	C Ins, 0.1%	-	Chi (WT >80%)
Plant12	10.6	0.99	WT, 88.7%	A Ins, 9.0%	T Ins, 0.8%	G Ins, 0.5%	-	-	Chi (WT >80%)
Plant13	62.5	0.98	A Ins, 59.8%	WT, 35.6%	G Ins, 2.0%	T Ins, 0.4%	-	-	Chi (WT ≤50%)
Plant14	0.6	0.99	WT, 98.5%	-	-	-	-	-	WT
Plant15	2.2	0.99	WT, 96.8%	A Ins, 1.1%	T Ins, 0.2%	-	-	-	Chi (WT >80%)
Plant16	1.7	0.97	WT, 95.8%	-	-	-	-	-	WT
Plant17	21.9	0.98	WT, 76.3%	A Ins, 16.6%	Del, 1.3%	T Ins, 1.1%	G Ins, 1.1%	-	Chi (50% <WT ≤80%)
Plant18	0.9	0.99	WT, 97.9%	-	-	-	-	-	WT
Plant19	9.8	0.99	WT, 89.2%	T Ins, 4.2%	A Ins, 3.8%	G Ins, 0.8%	-	-	Chi (WT >80%)
