# Supplementary material for: A screening method to identify efficient sgRNAs in Arabidopsis, used in conjunction with cell-specific lignin reduction
Source: Biotechnol Biofuels. 2019 May 23;12:130. doi: 10.1186/s13068-019-1467-y (PMC6532251; doi:10.1186/s13068-019-1467-y)
Supplement: Supplementary file 8 — Additional file 8. Mutation analysis of T1 transgenic plants of pUBQ10::CAS9-pU6::GONST2_gRNA1. [file 13068_2019_1467_MOESM8_ESM.pdf]

Additional File 8. Mutation analysis of T1 transgenic plants of pUBQ10::CAS9-pU6::GONST2_gRNA1. Genomic DNA was extracted from leaves of T1 Plant4 and Plant6. The region surrounding the target site of GONST2_gRNA1 was amplified and cloned. Twenty individual clones from each line were sequenced by Sanger sequencing.

Clone Name	Mutation Type
Plant4 Clone 1	46 bp Del[1]
Plant4 Clone 2	A Ins
Plant4 Clone 3	Mix of 46 bp Del[1] and A Ins
Plant4 Clone 4	7bp Ins[2]
Plant4 Clone 5	A Ins
Plant4 Clone 6	A Ins
Plant4 Clone 7	A Ins
Plant4 Clone 8	46 bp Del[1]
Plant4 Clone 9	A Ins
Plant4 Clone 10	A Ins
Plant4 Clone 11	A Ins
Plant4 Clone 12	A Ins
Plant4 Clone 13	A Ins
Plant4 Clone 14	A Ins
Plant4 Clone 15	Mix of 46 bp Del[1] and A Ins
Plant4 Clone 16	A Ins
Plant4 Clone 17	A Ins
Plant4 Clone 18	46 bp Del[1]
Plant4 Clone 19	A Ins
Plant4 Clone 20	A Ins
Plant6 Clone 1	19 bp Ins[3]
Plant6 Clone 2	19 bp Ins[3]
Plant6 Clone 3	T Insert
Plant6 Clone 4	Mix of T Ins and 19bp Ins[3]
Plant6 Clone 5	19 bp Ins[3]
Plant6 Clone 6	C Del
Plant6 Clone 7	C Del
Plant6 Clone 8	C Del
Plant6 Clone 9	19 bp Ins[3]
Plant6 Clone 10	T Insert
Plant6 Clone 11	not matching
Plant6 Clone 12	T Insert
Plant6 Clone 13	no priming
Plant6 Clone 14	T Insert
Plant6 Clone 15	C Del
Plant6 Clone 16	T Insert
Plant6 Clone 17	T Insert
Plant6 Clone 18	19 bp Ins[3]
Plant6 Clone 19	C Del
Plant6 Clone 20	not matching

[1]The 46 bp deletion sequence is: CGGTTGATGGAGATAACAGGCGTGACCACGGGTCTGTGAAACAGTC

[2]The 7bp insertion sequence is: AAATCTT

[3]The 19bp insertion sequence is: CAAATTGTCACAGGATAGA
