# Supplementary material for: A screening method to identify efficient sgRNAs in Arabidopsis, used in conjunction with cell-specific lignin reduction
Source: Biotechnol Biofuels. 2019 May 23;12:130. doi: 10.1186/s13068-019-1467-y (PMC6532251; doi:10.1186/s13068-019-1467-y)
Supplement: Supplementary file 9 — Additional file 9. Expression clones and their building parts. [file 13068_2019_1467_MOESM9_ESM.pdf]

Additional File 9. Expression clones and their building parts (For simplicity, terminators are not presented).

i) Expression vectors and parts for their assembly

**Construct Number**	**JBEI ICE Registry ID**	**Construct Name**	**Cloning Method**	**Construct Backbone**	**Integrated Piece(s)**
-	JBx_042298	pUBQ10	Acquired from JBEI Registry (Shih et al., 2016)	-	-
-	JBx_092437	pTKan-p35S::attR1-GW-attR2	Acquired from JBEI Registry (Gonzalez et al., 2015)	-	-
C11	JBx_092439	pTKan-pNST3::attR1-GW-attR2	Acquired from JBEI Registry	-	-
C50	JBx_092441	pTKan-pNOS::DsRed-p35S:: attR1-GW-attR2	Infusion	pTKan-p35S::attR1-GW-attR2, ApaI digest	pNOS:: DsRed
C381	JBx_092443	pTKan-pNOS::DsRed-p35S::PcoCAS9-AmpR- attR1-GW-attR2	Infusion	C50, AvrII digest	PcoCAS9 and AmpR
C382	JBx_092445	pTKan-pNOS::DsRed-pUBQ10::PcoCAS9-AmpR- attR1-GW-attR2	Infusion	C381, XhoI+AvrII digest	pUBQ10

ii) Expression clones and parts for their assembly

**Construct Number**	**JBEI ICE Registry ID**	**Construct Name**	**Cloning Method**	**Construct Backbone**	**Integrated Piece(s)**
C267	JBx_082113	pTKan-p35S::HCT::2A::Cys4-pNOS::cogGFP	Multisite Gateway	pTKan-p35S::attR1-GW-attR2	pDONR221-attL1-pU6:: HCT::2A::Cys4-attL4, pDONR221-attR4-pNOS-attR3, pDONR221-attL3-cogGFP-attL2
C349	JBx_092449	pTKan-pNST3::CAS9-pU6::HCT_gRNA12	Infusion	C11, AvrII+ HindIII digest	CAS9-pU6::HCT_gRNA12
C350	JBx_092451	pTKan-pNST3::CAS9-pU6::HCT_gRNA13	Infusion	C11, AvrII+ HindIII digest	CAS9-pU6::HCT_gRNA13
C351	JBx_092453	pTKan-pNST3::CAS9-pU6::HCT_gRNA14	Infusion	C11, AvrII+ HindIII digest	CAS9-pU6::HCT_gRNA14
C378	JBx_092455	pTKan-p35S::GONST2::2A::Cys4-pNOS::cogGFP	Infusion	C267, AscI digest	GONST2 (Jing et al., 2018)
C384	JBx_092457	pTKan-pNOS::DsRed-pUBQ10::PcoCAS9-AmpR-U6::Gonst2_gRNA1	Gateway	C382	pDONR/Zeo-attL1-pU6:: Gonst2_gRNA1-attL2
C385	JBx_070930	pTKan-pNOS::DsRed-pUBQ10::PcoCAS9-AmpR-U6::Gonst2_gRNA2	Gateway	C382	pDONR/Zeo-attL1-pU6:: Gonst2_gRNA2-attL2
C409	JBx_076699	pTKan-pNOS::DsRed-pUBQ10::PcoCAS9-AmpR-pU6::HCT_gRNA1	Gateway	C382	pDONR/Zeo-attL1-pU6::HCT_gRNA1-attL2
C410	JBx_076701	pTKan-pNOS::DsRed-pUBQ10::PcoCAS9-AmpR-pU6::HCT_gRNA2	Gateway	C382	pDONR/Zeo-attL1-pU6::HCT_gRNA2-attL2
C412	JBx_076705	pTKan-pNOS::DsRed-pUBQ10::PcoCAS9-AmpR-pU6::HCT_gRNA3	Gateway	C382	pDONR/Zeo-attL1-pU6::HCT_gRNA3-attL2
C413	JBx_076707	pTKan-pNOS::DsRed-pUBQ10::PcoCAS9-AmpR-pU6::HCT_gRNA4	Gateway	C382	pDONR/Zeo-attL1-pU6::HCT_gRNA4-attL2
C414	JBx_076159	pTKan-pNOS::DsRed-pUBQ10::PcoCAS9-AmpR-pU6::HCT_gRNA5	Gateway	C382	pDONR/Zeo-attL1-pU6::HCT_gRNA5-attL2
C415	JBx_076709	pTKan-pNOS::DsRed-pUBQ10::PcoCAS9-AmpR-pU6::HCT_gRNA6	Gateway	C382	pDONR/Zeo-attL1-pU6::HCT_gRNA6-attL2
C416	JBx_076711	pTKan-pNOS::DsRed-pUBQ10::PcoCAS9-AmpR-pU6::HCT_gRNA7	Gateway	C382	pDONR/Zeo-attL1-pU6::HCT_gRNA7-attL2
C417	JBx_076161	pTKan-pNOS::DsRed-pUBQ10::PcoCAS9-AmpR-pU6::HCT_gRNA8	Gateway	C382	pDONR/Zeo-attL1-pU6::HCT_gRNA8-attL2
C418	JBx_076163	pTKan-pNOS::DsRed-pUBQ10::PcoCAS9-AmpR-pU6::HCT_gRNA9	Gateway	C382	pDONR/Zeo-attL1-pU6::HCT_gRNA9-attL2
C419	JBx_076165	pTKan-pNOS::DsRed-pUBQ10::PcoCAS9-AmpR-pU6::HCT_gRNA10	Gateway	C382	pDONR/Zeo-attL1-pU6::HCT_gRNA10-attL2
C420	JBx_076167	pTKan-pNOS::DsRed-pUBQ10::PcoCAS9-AmpR-	Gateway	C382	pDONR/Zeo-attL1-pU6::HCT_gRNA11-attL2

		pU6::HCT_gRNA11			
C421	JBx_076169	pTKan-pNOS::DsRed-pUBQ10::PcoCAS9-AmpR-pU6::HCT_gRNA12	Gateway	C382	pDONR/Zeo-attL1-pU6::HCT_gRNA12-attL2
C422	JBx_076171	pTKan-pNOS::DsRed-pUBQ10::PcoCAS9-AmpR-pU6::HCT_gRNA13	Gateway	C382	pDONR/Zeo-attL1-pU6::HCT_gRNA13-attL2
C423	JBx_076713	pTKan-pNOS::DsRed-pUBQ10::PcoCAS9-AmpR-pU6::HCT_gRNA14	Gateway	C382	pDONR/Zeo-attL1-pU6::HCT_gRNA14-attL2

GONZALEZ, T. L., LIANG, Y., NGUYEN, B. N., STASKAWICZ, B. J., LOQUÉ, D. & HAMMOND, M. C. 2015. Tight regulation of plant immune responses by combining promoter and suicide exon elements. *Nucleic Acids Research,* 43**,** 7152-7161.

JING, B., ISHIKAWA, T., SOLTIS, N., INADA, N., LIANG, Y., MURAWSKA, G., ANDEBERHAN, F., PIDATALA, V., YU, X., BAIDOO, E., KAWAI-YAMADA, M., LOQUE, D., KLIEBENSTEIN, D., DUPREE, P. & MORTIMER, J. 2018. GONST2 transports GDP-Mannose for sphingolipid glycosylation in the Golgi apparatus of Arabidopsis. *bioRxiv*.

SHIH, P. M., VUU, K., MANSOORI, N., AYAD, L., LOUIE, K. B., BOWEN, B. P., NORTHEN, T. R. & LOQUE, D. 2016. A robust gene-stacking method utilizing yeast assembly for plant synthetic biology. *Nat Commun,* 7**,** 13215.
