# Supplementary material for: A screening method to identify efficient sgRNAs in Arabidopsis, used in conjunction with cell-specific lignin reduction
Source: Biotechnol Biofuels. 2019 May 23;12:130. doi: 10.1186/s13068-019-1467-y (PMC6532251; doi:10.1186/s13068-019-1467-y)
Supplement: Supplementary file 10 — Additional file 10. Sequence of the synthetic parts. [file 13068_2019_1467_MOESM10_ESM.pdf]

Additional File 10. Sequences of synthetic parts.

> pU6::HCT_gRNA1::tNOS

gacaagtttgtacaaaaaagcaggcttcGGAGTGATCAAAAGTCCCACATCGATCAGGTGATATATAGCAGCTTAGTTTATA
TAATGATAGAGTCGACATAGCGATT**GTCGCTTGAAGAGAGACGATGA**GTTTTAGAGCTAGAAATAGCAAGTTAA
AATAAGGCTAGTCCGTTATCAACTTGAAAAAGTGGCACCGAGTCGGTGCTTTTTTTTcctaggTTCAAACATTTGGCA
ATAAAGTTTCTTAAGATTGAATCCTGTTGCCGGTCTTGCGATGATTATCATATAATTTCTGTTGAATTACGTTAAGCA
TGTAATAATTAACATGTAATGCATGACGTTATTTATGAGATGGGTTTTTATGATTAGAGTCCCGCAATTATACATTT
AATACGCGgacccagctttcttgtacaaagtggtc

HCT_gRNA1 spacer sequence is shown in bold; U6 promoter sequence is shown in red; sgRNA scaffold sequence is shown in blue; NOS terminator sequence is shown in black; attB1 and attB2 sites are shown in lower case letters on 5' end and 3' end of the sequence.

> HCT::fsSTOP::2A::Cys4

ggacaagtttgtacaaaaaagcaggcttcATGTTAATTAACGGCGCGCCAAAAATTAACATCAGAGATTCCACCATGGTCCG
GCCTGCCACCGAGACACCAATCACTAATCTTTGGAACTCCAACGTCGACCTTGTCATCCCCAGATTCCATACCCCTA
GTGTCTACTTCTACAGACCCACCGGCGCTTCCAATTTCTTTGACCCTCAGGTCATGAAGGAAGCTCTTTCCAAAGCC
CTTGTCCCTTTTTACCCTATGGCTGGTCGCTTGAAGAGAGACGATGATGGTCGTATTGAGATCGATTGTAACGGTG
CTGGTGTTCTCTTCGTTGTGGCTGATACTCCTTCTGTTATCGATGATTTTGGTGATTTTGCTCCTACCCTTAATCTCCG
TCAGCTTATTCCCGAAGTTGATCACTCCGCTGGCATTCACTCTTTCCCGCTTCTCGTTTTGCAGGTGACTTTCTTTAA
ATGTGGGGGAGCTTCACTTGGGGTTGGGATGCAACATCACGCGGCAGATGGTTTCTCTGGTCTTCATTTTATCAAC
ACATGGTCTGATATGGCTCGTGGTCTTGACCTAACCATTCCACCTTTCATTGATCGAACACTCCTCCGAGCTAGGGA
CCCGCCACAGCCTGCTTTTCATCATGTTGAATATCAGCCTGCACCAAGTATGAAGATACCTCTTGATCCGTCTAAAT
CAGGACCTGAGAATACCACTGTCTCTATATTCAAATTAACACGAGACCAGCTTGTTGCTCTTAAGGCGAAATCCAA
GGAGGATGGGAACACTGTCAGCTACAGCTCATACGAGATGTTGGCAGGGCATGTGTGGAGATCAGTGGGAAAG
GCGCGAGGGCTTCCAAACGACCAAGAGACGAAACTGTACATTGCAACTGATGGAAGGTCTAGACTACGTCCGCAG
CTGCCTCCTGGTTACTTTGGGAATGTGATATTCACTGCAACACCATTGGCTGTTGCAGGGGATTTGTTATCTAAGCC
AACATGGTATGCTGCAGGACAGATTCATGATTTCTTGGTTCGTATGGATGATAACTATCTGAGGTCAGCTCTTGAC
TACCTGGAGATGCAGCCTGATCTGTCAGCCCTTGTCCGCGGTGCACATACCTACAAGTGCCCAAATTTGGGAATCA
CAAGCTGGGTTAGATTACCTATTTATGATGCAGACTTTGGTTGGGGTCGTCCTATCTTTATGGGACCTGGTGGAAT
TCCATACGAGGGTTTGTCTTTTGTGCTACCAAGTCCTACTAATGATGGCAGCTTATCCGTTGCCATTGCCCTCCAATC
TGAACACATGAAACTGTTTGAGAAGTTTTTGTTTGAGATAGCGCTTCAAGGTGCACATGGAGGGGGCGCGCCACG
TGATCTAGGCCGTCAGCTGTTGAATTTTGACCTTCTTAAGCTTGCGGGAGACGTCGAGTCCAACCCTGGGCCCATG
GCTCCAAAAAAGAAAAGAAAGGTTGCTAGCGACCATTACTTAGATATTAGATTGAGACCAGACCCAGAGTTCCCTC
CAGCACAGTTGATGTCAGTGTTATTCGGAAAATTGCACCAAGCTTTGGTTGCACAAGGTGGAGATAGAATTGGTG
TGAGTTTTCCTGATTTGGACGAGTCTAGGTCAAGATTGGGAGAAAGGTTAAGAATCCATGCTTCAGCAGATGACCT
TAGGGCTTTGTTAGCAAGACCTTGGTTAGAGGGTCTTAGAGATCATTTGCAGTTCGGAGAACCAGCTGTTGTGCCA
CACCCTACACCATATAGACAAGTTTCTAGAGTGCAGGCAAAATCAAATCCTGAGAGACTTAGAAGGAGATTGATG
AGGAGACACGACTTATCTGAAGAGGAAGCTAGGAAGAGAATACCTGATACCGTTGCTAGAGCACTTGACTTGCCA
TTTGTGACTTTGAGAAGTCAATCCACTGGTCAGCATTTTAGGTTATTCATTAGACACGGACCACTTCAAGTTACTGC
AGAGGAGGGAGGATTCACCTGTTATGGATTGAGTAAGGGAGGATTCGTGCCTTGGTTCGATCCTGCATTTCTTTAT
AAGGTTGTTTGAcacccaacttttctatacaaagttgtcccc

HCT gene is shown in yellow; a linker sequence containing a frameshifted stop codon cassette is shown in grey; potential stop codons being activated by genome editing in preceding sequences

are underlined; 2A peptide is shown in blue; Csy4 with nuclear localization signal is shown in orange; attB2 tag for immunoblotting is shown in black; attB1 and attB4 sites are shown in lower case letters on 5' end and 3' end of the sequence.
