# Supplementary material for: A screening method to identify efficient sgRNAs in Arabidopsis, used in conjunction with cell-specific lignin reduction
Source: Biotechnol Biofuels. 2019 May 23;12:130. doi: 10.1186/s13068-019-1467-y (PMC6532251; doi:10.1186/s13068-019-1467-y)
Supplement: Supplementary file 11 — Additional file 11. Primer list. [file 13068_2019_1467_MOESM11_ESM.pdf]

Additional File 11. Primer list

Name	Experiment, 5'->3' sequence	Tm
Fragments for Infusion Cloning of C50		
pNOS::DsRed::tNOS		
F-pTKan-ApaIe-pNOS	ggggatcctctagagGATACATGAGAATTAAGGGAG	
R-tNOS-pTKan-ApaI	tccgcggacgtcccgggcccGAGCTTGCATGCCGGTCG	
Fragments for Infusion Cloning of C381		
PcoCAS9		59
pTKanAvrIICACCPcoCAS9_F	gaacacgggggactcctaggCACCatggattacaaggatg	
tG7AscICAS9	gctcatcctagttagtcagtGGCGCGCctacttcttcttcttagcctgtcc	
tG7-AmpR		53
CAS9AscItG7_F	GCGCGCCactgactaactaggatgagc	
pTKanavrIIdAmp_R	ataacaggctagccctagttaccaatgcttaatcagtgag	
Fragments for Infusion Cloning of C382		
pUBQ10		57
XhoIpUBQ10_F	ggagatctacgcgtgtcgactcgagggagtacccgacgagtcag	
UBQ10AvrII_R	catccttgtaatccatggtgcctaggagtgttaatcagaaaaactcag	
Fragments for Infusion Cloning of pENTR Vectors with Various sgRNA		
Piece1 for entry vector with various spacer sequence		58
Piece1F (pairing with gRNAspecific_R)	ccagtcttaagctcgggccc	
Piece2 for entry vector with various spacer sequence		50
Piece2R (pairing with gRNAspecific_F)	tacgactcactataggggatatc	
Piece3 for entry vector with various spacer sequence		
Piece3F	gatatcccctatagtgagtcg	57
Piece3R	ggcccgagcttaagactg	63
HCT_gRNA2 specific primers for entry vector		
HCT_gRNA2F	GTCTACTTCTACAGACCCACgttttagagctagaaatagc	
HCT_gRNA2R	GTGGGTCTGTAGAAGTAGACaatcgctatgtcgactctatc	
HCT_gRNA3 specific primers for entry vector		
HCT_gRNA3F	GGTATGTGCACCGCGGACAAgttttagagctagaaatagc	
HCT_gRNA3R	TTGTCCGCGGTGCACATACCaatcgctatgtcgactctatc	
HCT_gRNA4 specific primers for entry vector		
HCT_gRNA4F	GCTGTGGCGGGTCCCTAGCTgttttagagctagaaatagc	
HCT_gRNA4R	AGCTAGGGACCCGCCACAGCaatcgctatgtcgactctatc	
HCT_gRNA5 specific primers for entry vector		
HCT_gRNA5F	GACAGTGTTCCCATCCTCCTgttttagagctagaaatagc	
HCT_gRNA5R	AGGAGGATGGGAACACTGTCaatcgctatgtcgactctatc	
HCT_gRNA6 specific primers for entry vector		
HCT_gRNA6F	GAGGTCAGCTCTTGACTACCgttttagagctagaaatagc	
HCT_gRNA6R	GGTAGTCAAGAGCTGACCTCaatcgctatgtcgactctatc	
HCT_gRNA7 specific primers for entry vector		
HCT_gRNA7F	GATAAGCTGCCATCATTAGTgttttagagctagaaatagc	
HCT_gRNA7R	ACTAATGATGGCAGCTTATCaatcgctatgtcgactctatc	
HCT_gRNA8 specific primers for entry vector		
HCT_gRNA8F	GATATTCACTGCAACACCATgttttagagctagaaatagc	
HCT_gRNA8R	ATGGTGTTGCAGTGAATATCaatcgctatgtcgactctatc	
HCT_gRNA9 specific primers for entry vector		
HCT_gRNA9F	GATCAGTGGGAAAGGCGCGAgttttagagctagaaatagc	

HCT_gRNA9R	TCGCGCCTTTCCCACTGATCaatcgctatgtcgactctatc	
HCT_gRNA10 specific primers for entry vector		
HCT_gRNA10F	GATCTGTCAGCCCTTGTCCGgttttagagctagaaatagc	
HCT_gRNA10R	CGGACAAGGGCTGACAGATCaatcgctatgtcgactctatc	
HCT_gRNA11 specific primers for entry vector		
HCT_gRNA11F	GATGAAAAGCAGGCTGTGGCgttttagagctagaaatagc	
HCT_gRNA11R	GCCACAGCCTGCTTTTCATCaatcgctatgtcgactctatc	
HCT_gRNA12 specific primers for entry vector		
HCT_gRNA12F	GCTCATACGAGATGTTGGCAgttttagagctagaaatagc	
HCT_gRNA12R	TGCCAACATCTCGTATGAGCaatcgctatgtcgactctatc	
HCT_gRNA13 specific primers for entry vector		
HCT_gRNA13F	GTATGGATGATAACTATCTGgttttagagctagaaatagc	
HCT_gRNA13R	CAGATAGTTATCATCCATACaatcgctatgtcgactctatc	
HCT_gRNA14 specific primers for entry vector		
HCT_gRNA14F	GTGTTCAGATTGGAGGGCAAgttttagagctagaaatagc	
HCT_gRNA14R	TTGCCCTCCAATCTGAACACaatcgctatgtcgactctatc	
GONST2_gRNA1 specific primers for entry vector		
GONST2_gRNA1F	GAGATAACAGGCGTGACCACgttttagagctagaaatagc	
GONST2_gRNA1R	GTGGTCACGCCTGTTATCTCaatcgctatgtcgactctatc	
GONST2_gRNA2 specific primers for entry vector		
GONST2_gRNA2F	GTTTGGTGGGTTCATTAAACAgttttagagctagaaatagc	
GONST2_gRNA2R	TGTTTAATGAACCCACCAAACaatcgctatgtcgactctatc	
Sequences franking HCT_gRNA12 in tobacco		63
HCTsite601F	gaatatcagcctgcaccaag	
HCTsite900R	CACATTCCCAAAGTAACCAGG	
Sequences franking HCT_gRNA14 in tobacco		62
HCTsite1157F	ctttatgggacctggtggaattc	
CSY4site11R	aagtaatggtcgctagcaacc	
Sequences franking HCT_gRNA14 in Arabidopsis		
HCTsite1066F	ggtgcacatacctacaagtgc	62
HCT3'UTR230_R	catacagcatggtggatataacc	
Sequences franking Gonst2_gRNA1		
GONST2-102F	tctttcaacttctcgatcag	60
GONST2-578R	AGATATCTAGTACAGCTACCAC	
Sequences franking Gonst2_gRNA2		60
GONST2-1684F	GGTAGTGGCAACAGCAAG	
GONST2 R2	TGACATTTTAGCTCTGGCAAAG	
