# Supplementary material for: A screening method to identify efficient sgRNAs in Arabidopsis, used in conjunction with cell-specific lignin reduction
Source: Biotechnol Biofuels. 2019 May 23;12:130. doi: 10.1186/s13068-019-1467-y (PMC6532251; doi:10.1186/s13068-019-1467-y)
Supplement: Supplementary file 12 — Additional file 12. A map demonstrating In-Fusion cloning of Entry Clones containing individual sgRNAs. [file 13068_2019_1467_MOESM12_ESM.pdf]

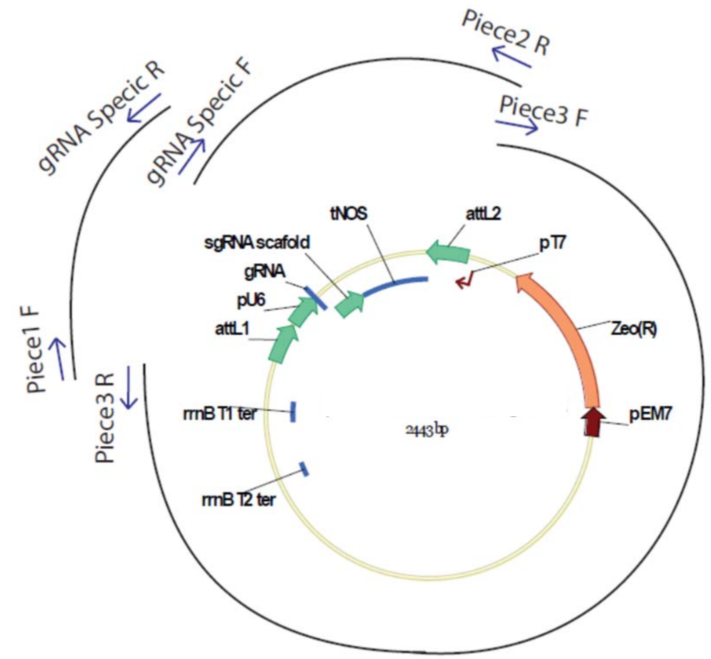

Additional File 12. A map demonstrating In-Fusion cloning of Entry Clones containing individual sgRNAs.
